# Chronic immunosuppression across 12 months and high ability of acute and subacute CNS-injury biomarker concentrations to identify individuals with complicated mTBI on acute CT and MRI

**DOI:** 10.1186/s12974-024-03094-8

**Published:** 2024-04-27

**Authors:** Gerard Janez Brett Clarke, Turid Follestad, Toril Skandsen, Henrik Zetterberg, Anne Vik, Kaj Blennow, Alexander Olsen, Asta Kristine Håberg

**Affiliations:** 1 Department of Radiology and Nuclear Medicine, St. Olavs Hospital, Trondheim University Hospital, Trondheim, Norway; 2 Department of Neuromedicine and Movement Sciences, NTNU, Trondheim, Norway; 3 Department of Clinical and Molecular Medicine, Norwegian University of Science and Technology (NTNU), Trondheim, N-7491 Norway; 4 Clinic of Rehabilitation, St. Olavs Hospital, Trondheim University Hospital, Trondheim, Norway; 5 Department of Psychiatry and Neurochemistry, Institute of Neuroscience and Physiology, Sahlgrenska Academy, University of Gothenburg, Mölndal, Sweden; 6 Clinical Neurochemistry Laboratory, Sahlgrenska University Hospital, Mölndal, Sweden; 7 Department of Neurodegenerative Disease, UCL Queen Square Institute of Neurology, Queen Square, London, UK; 8 UK Dementia Research Institute at UCL, London, UK; 9 Hong Kong Center for Neurodegenerative Diseases, Clear Water Bay, Sha Tin, Hong Kong China; 10 Wisconsin Alzheimer’s Disease Research Center, University of Wisconsin School of Medicine and Public Health, University of Wisconsin-Madison, Madison, WI USA; 11 Department of Neurosurgery, St Olavs Hospital, Trondheim University Hospital, Trondheim, Norway; 12 Department of Psychology, Norwegian University of Science and Technology, Trondheim, Norway; 13 NorHEAD - Norwegian Centre for Headache Research, Trondheim, Norway

**Keywords:** Concussion, Mixed-mechanism mild TBI, Prediction, Predictive modeling, Cytokines, Growth factors, Neuroimaging

## Abstract

**Background:**

Identifying individuals with intracranial injuries following mild traumatic brain injury (mTBI), i.e. complicated mTBI cases, is important for follow-up and prognostication. The main aims of our study were (1) to assess the temporal evolution of blood biomarkers of CNS injury and inflammation in individuals with complicated mTBI determined on computer tomography (CT) and magnetic resonance imaging (MRI); (2) to assess the corresponding discriminability of both single- and multi-biomarker panels, from acute to chronic phases after injury.

**Methods:**

Patients with mTBI (*n* = 207), defined as Glasgow Coma Scale score between 13 and 15, loss of consciousness < 30 min and post-traumatic amnesia < 24 h, were included. Complicated mTBI – i.e., presence of any traumatic intracranial injury on neuroimaging – was present in 8% (*n* = 16) on CT (CT+) and 12% (*n* = 25) on MRI (MRI+). Blood biomarkers were sampled at four timepoints following injury: admission (within 72 h), 2 weeks (± 3 days), 3 months (± 2 weeks) and 12 months (± 1 month). CNS biomarkers included were glial fibrillary acidic protein (GFAP), neurofilament light (NFL) and tau, along with 12 inflammation markers.

**Results:**

The most discriminative single biomarkers of traumatic intracranial injury were GFAP at admission (CT+: AUC = 0.78; MRI+: AUC = 0.82), and NFL at 2 weeks (CT+: AUC = 0.81; MRI+: AUC = 0.89) and 3 months (MRI+: AUC = 0.86). MIP-1β and IP-10 concentrations were significantly lower across follow-up period in individuals who were CT+ and MRI+. Eotaxin and IL-9 were significantly lower in individuals who were MRI+ only. FGF-basic concentrations increased over time in MRI- individuals and were significantly higher than MRI+ individuals at 3 and 12 months. Multi-biomarker panels improved discriminability over single biomarkers at all timepoints (AUCs > 0.85 for admission and 2-week models classifying CT+ and AUC ≈ 0.90 for admission, 2-week and 3-month models classifying MRI+).

**Conclusions:**

The CNS biomarkers GFAP and NFL were useful single diagnostic biomarkers of complicated mTBI, especially in acute and subacute phases after mTBI. Several inflammation markers were suppressed in patients with complicated versus uncomplicated mTBI and remained so even after 12 months. Multi-biomarker panels improved diagnostic accuracy at all timepoints, though at acute and 2-week timepoints, the single biomarkers GFAP and NFL, respectively, displayed similar accuracy compared to multi-biomarker panels.

**Supplementary Information:**

The online version contains supplementary material available at 10.1186/s12974-024-03094-8.

## Introduction

Mild traumatic brain injury (mTBI) is the most common type of brain injury, representing up to 90% of all cases of traumatic brain injury [1, 2]. It encompasses a wide range of injury severities, from blows to the head with limited symptoms and rapid recovery, to injuries involving intracranial abnormalities detectable with neuroimaging techniques. Individuals with mTBI concurrent with traumatic intracranial findings determined by computed tomography (CT) or magnetic resonance imaging (MRI) are considered to have experienced a complicated mTBI. These individuals are at increased risk for cognitive sequalae and persistent post-concussive symptoms [3–6]. Given this, it is important to identify patients with complicated mTBI who are at higher risk of post-injury complications.

CT is currently the mainstay imaging technique used in the acute care of patients with TBI [6–8], yet there is a growing concern regarding the overuse of CT in mTBI diagnostics, due to unnecessary radiation exposure and high cost [9]. Low-cost and reliable identification of patients with potential intracranial injury in the emergency department (ED), through blood-based biomarkers, could drastically improve patient triage protocols. Two biomarkers: UCH-L1 and GFAP are currently approved by the Food and Drug Administration in the US to assess the likelihood of mTBI-related intracranial injury in the acute phase [10]. In Scandinavia, blood S100B is recommended for triaging patients with mTBI to CT scanning during the first 24 h after injury [11]. Despite this, it remains unclear whether other blood biomarkers, or a combination of such biomarkers could improve diagnostic accuracy in the acute phase.

Moreover, MRI is known to be more sensitive for detecting certain brain injuries than CT, namely in identifying traumatic axonal injury (TAI) – including microbleeds [8, 12, 13] – that are difficult to observe on CT [3–5]. There are currently no recognized guidelines for determining which patients should be referred to clinical MRI examination instead of CT [6–8], although emerging evidence suggests outcome prediction is improved based on MRI findings compared to CT [12], and many patients with no observable lesions on CT have findings on MRI [14].

It also remains to be shown whether a biomarker – or panel of biomarkers – sampled during subacute and late/chronic phases could be diagnostically linked to acute phase traumatic intracranial findings. Since most acute intracranial injuries following mTBI resolve over time [15], blood biomarkers able to reliably identify patients with complicated mTBI at later timepoints could be of great diagnostic utility for patients who present to the clinic long after initial injury. This could prove helpful also for individuals with mTBI who are involved in litigation. Neurofilament light (NFL) – considered a surrogate marker for axonal injury [16] – is a promising candidate in this regard, given its late peak (∼ 10 days after injury) [17], and evidenced protracted course of elevation for at least 3 months in mTBI [18] (and up to 5 years following moderate-severe TBI) [19]. Recent studies have suggested acutely measured NFL is able to discriminate intracranial abnormalities in patients with mTBI on both CT and MRI [20–22], though its diagnostic utility at later timepoints is yet to be determined.

Lastly, neuroinflammation is a known acute pathophysiological consequence of TBI, with a clearly evidenced duality of both harmful and beneficial secondary effects [23, 24]. So far, research on TBI and inflammatory mechanisms has been primarily undertaken in animal models and on moderate-severe TBI cohorts in humans [24, 25]. Recent work on mild TBI cohorts has evidenced associations between certain inflammation markers and complicated mTBI (determined by both CT and MRI) [22, 26–28]. However, many markers of inflammation in the context of mTBI diagnosis remain unexplored, as studies have typically pre-selected a small number of inflammation markers for analysis, and only during the acute phase after injury. This highlights the need for investigations into the temporal evolution of mTBI-associated inflammation, including whether there are dissociable inflammatory profiles in those with acute intracranial injury on CT/MRI compared to those without.

Our study’s aims are two-fold, and intended to contribute to the goals of precision-based medicine to generate more personalized treatment protocols [29]:

1. To assess the longitudinal evolution of a large array of biomarkers related to peripheral inflammation and CNS damage over the course of one year following injury in patients with complicated versus uncomplicated mild TBI, as determined by CT (performed within 24 h) and MRI (performed within 72 h). Patients with mTBI were followed up with blood sampled at four timepoints after injury: admission (within 72 h), 2 weeks (± 3 days), 3 months (± 2 weeks) and 12 months (± 1 month).
2. To assess the ability of single and multiple blood biomarkers (i.e. a multi-biomarker panel) to classify those with complicated mTBI based on CT (CT+) and MRI (MRI+), compared to those without (CT-/MRI-), at each of the four timepoints. Model classification accuracy was assessed with area under the curves (AUCs) and multiple biomarker selection was performed using elastic net methodology. Further to this, we aimed to establish whether there are any biomarker profiles uniquely associated with findings on MRI vs. CT, thereby uncovering potential diagnostic biomarkers that could aid in triaging patients to MRI instead of (or in addition to) CT.

## Materials and methods

### Participants and recruitment

The Trondheim mTBI study is a large-scale prospective cohort study with follow up for 12 months in patients with mTBI between 16 and 60 years of age. Patients with mTBI (*n* = 378) were included from April 1st 2014 to December 15th 2015. They were recruited from two emergency departments (EDs): St. Olavs hospital (Trondheim University Hospital), a regional level 1 trauma center in Trondheim, Norway, and Trondheim Municipal Emergency clinic, a general practitioner-run, 24-hour/7-day out-patient clinic.

Inclusion criteria were having sustained a mild TBI according to World Health Organization criteria [30], i.e. Glasgow Coma Scale (GCS) score of 13–15, < 30 min loss of consciousness (LOC), and < 24 h post-traumatic amnesia (PTA). Exclusion criteria were: (1) non-fluency in the Norwegian language, (2) pre-existing neurological, psychiatric, somatic, or substance use disorder; determined to be severe enough to interfere with follow-up and outcome assessment, (3) a prior history of a complicated mild (i.e. having trauma-related intracranial findings on CT or MRI), moderate or severe TBI, (4) other major trauma that could interfere with follow-up or outcome assessment, or (5) presentation > 48 h after the initial trauma. The sub-cohort selected for this investigation were all patients with mTBI (see Skandsen et al. [31] and Einarsen et al. [14] for more details regarding patient enrolment and clinical ratings) who had blood data collected.

### Clinical information

Clinical information was obtained from patient interviews and medical records. LOC was rated as present only if observed. Duration of PTA was recorded as time after injury for which the patient had no continuous memory (> 0 min and < 1 h, or 1–24 h). GCS score was assessed in the ED or inferred from records [32]. Presence of injuries to parts of the body other than the head (e.g. dislocations, fractures, soft tissue injuries in need of treatment) was recorded based on self-report and ED/hospital records. Skin abrasions and contusions were not included in this rating.

### CT imaging

Non-contrast CT was performed on a Siemens Somatom Sensation 64 row scanner as part of the initial clinical assessment (within 24 h of injury), according to the then Scandinavian Guidelines for Initial Management of Minimal, Mild and Moderate Head Injuries [33]. The intracranial traumatic findings were classified by an experienced neuroradiologist into contusion, epidural hematoma (EDH), traumatic sub-arachnoid hemorrhage (tSAH) and subdural hematoma (SDH). Presence of any of these findings in a patient led to their classification into the CT + group used in analyses. The CT scans from patients with intracranial traumatic findings on MRI were later reviewed by an experienced neuroradiologist and a consultant in physical medicine and rehabilitation.

### MRI imaging

Subjects underwent a standardized brain MRI scan within 72 h of injury [31]. All MRI scans were acquired with the same protocol on the same 3.0 Tesla Siemens Skyra MRI scanner with a 32-channel head coil. The protocol included 3D volumes with T1-weighted (Magnetization Prepared Rapid Acquisition Gradient Echo), T2-weighted, Fluid-attenuated inversion recovery (FLAIR), and susceptibility-weighted (SWI) scans. The clinical scans were read by neuroradiologists according to standard criteria [14], and the inter-rater reliability was moderate to good. In addition to determining contusions and hematomas, TAI was diagnosed and graded as described previously [34]. Presence of any of these findings in a patient led to their classification into the MRI + group used in analyses. Two patients with a positive CT scan were unable to undergo MRI at inclusion, hence the reading of the CT scan was used to describe TBI-related intracranial findings in place of MRI. More detailed patient MRI results and their development over time are presented in Einarsen et al. [14].

### Blood samples

Time of blood sampling was measured as time from injury. Participants had their blood drawn at admission (within 72 h post-injury), then at 2 weeks (± 3 days), 3 months (± 2 weeks), and 12 months (± 1 month). The admission sample was drawn either in the ER (within 24 h of injury), or at time of MRI scan (within 72 h of injury). Plasma samples were obtained with EDTA gel tubes which were immediately put on ice and centrifuged for 10 min at 4 °C on 2,000 g within 30 min of acquisition and aliquoted into eight 0.5 mL Nunc tubes which were immediately frozen at -80 °C. The tubes remained stored at -80 °C until two tubes were retrieved and transported in the frozen condition to the labs that analyzed the CNS injury and the inflammation makers, respectively. No freeze thaw cycles were necessary.

Plasma GFAP, NFL and tau concentrations were measured using the validated and commercially available Human Neurology 4-Plex A assay (N4PA) on an HD-1 Single molecule array (Simoa) instrument, according to instructions from the manufacturer (Quanterix, Billerica, MA). The measurements were performed in one round of experiments using one batch of reagents by board-certified laboratory technicians blinded to the clinical data. See Clarke et al. [35] for further details.

For inflammation markers, the plasma samples were analyzed using a commercial fluorescence magnetic bead-based immunoassay, with high-sensitivity detection range and precision (Bio-Plex Human Cytokine 27-Plex, Bio-Rad Laboratories, Inc., Hercules, CA, USA). 27 inflammation markers were analyzed in total (see Chaban et al. [36] for full list). This panel was chosen as it represents a comprehensive selection of the most common markers of inflammation. Plasma samples were diluted 1:4 in Sample Diluent (Bio-Rad Laboratories, Inc.). A lower detection limit for the cytokines in the low picogram/milliliter range (< 20 pg/mL for all cytokines) was determined automatically by the software based on the standard curve for each inflammation marker. Only markers that were present in methodologically and clinically meaningful amounts, according to our previous experience [37], in more than 75% of all samples during the observation period, were selected for further study. These were: IL-1 receptor antagonist (IL-1ra), IL-8, IL-9, IL-17 A, eotaxin-1 (CCL11), basic fibroblast growth factor (FGF-basic), interferon gamma (IFN-γ), IFN-γ-inducing protein 10 (IP-10/CXCL10), monocyte chemoattractant protein 1 (MCP-1/CCL2), macrophage inflammatory protein-1-beta (MIP-1β/CCL4), platelet-derived growth factor-BB (PDGF-BB), tumor necrosis factor (TNF).

### Statistical analysis

Demographic and clinical variables for the total number of patients included in this study are summarized by frequencies and percentages or means and standard deviations, as appropriate. Descriptive statistics (mean, standard deviation, median, interquartile range and range) for biomarkers are presented per timepoint in supplementary Tables 1 and 2.

Linear mixed model (LMM) analyses were conducted to assess the temporal evolution of biomarkers, with grouping variables separated by CT+/CT- and MRI+/MRI- and biomarkers as outcomes. Time (reflecting the four timepoints of blood sampling), group, and a time-by-group interaction were entered as fixed effects. Interactions were retained in all models regardless of statistical significance. The biomarkers previously determined to deviate from normality (GFAP, NFL, tau, IL-1ra, eotaxin, MCP-1, and IP-10) [18, 36], were base-10 log-transformed prior to inclusion in the model. To account for within-subject correlations, a covariance structure for the total residuals was selected among a set of candidate models: (1) a model with an unstructured correlation matrix and homogeneous residual variance (UC-model), (2) a random intercept only model (RI-model), and (3) a random intercept model with heterogenous residual variances (HV-model). A fully unstructured covariance structure, including heterogeneous variances, was ruled out due to lack of convergence of the fitting algorithm. Model fit was assessed using a pragmatic combination of Aikake information criterion (AIC) and log-likelihood ratio (LR) tests, aimed at selecting the most parsimonious model with an acceptable model fit (without considering a specific threshold of significance).

Main effects are presented in supplementary Table 3. For biomarkers showing a significant group effect or a significant time-by-group interaction, group differences at each timepoint were assessed using post-hoc contrasts adjusted by Tukey’s honest significant difference (HSD). Within-group changes across time were assessed only if there was a significant time-by-group interaction. Only significant effects of group (CT+/CT- or MRI+/MRI-) or a time by group interaction were of interest, as pure effects of time in the mTBI group as a whole have been previously reported on [18]. Separate models were generated with sex and sex-by-group interaction as potential covariates of interest (main effects presented in supplementary Table 4). There was a significant effect of sex only on tau, without a significant interaction between sex and group. As this effect has been previously reported on [18], and no other significant sex effects were present, sex effects were not included in the analyses presented in the results section.

To determine the best combination of biomarkers for predicting patients who were CT + and MRI+, elastic net regression was performed using all candidate biomarkers at each timepoint as possible predictors. Elastic net models are generalized linear models fit with a hybrid of lasso and ridge penalty functions [38]. Ridge regression penalizes the square of the regression coefficients for the predictors, shrinking coefficients for the least important predictors toward zero. Lasso imposes a penalty on the absolute value of the coefficients, shrinking them by a constant factor, thereby selecting a subset of predictors by shrinking the coefficients of the least predictive predictors to zero. Whereas ridge retains all predictors, adjusting for relative predictive importance, lasso tends to select only one predictor from a group of correlated predictors. Elastic net is a useful combination of both, performing shrinkage selection while enabling the inclusion of collinear predictors in the final model. This means all variables that have a meaningful effect on the outcome can be selected by the procedure, even if they are strongly correlated, while predictors unrelated to outcome will be set to 0.

To determine the optimal penalization parameters and internally validate models, 5-fold cross-validation (CV) was used, testing over a grid of α and λ sequences and selecting the combination yielding the maximal AUC value. Uncertainty in variable selection was assessed by repeating the penalized regression procedure for each model in 1000 bootstrap samples. The uncertainty for each of the variables was assessed as the proportion of the 1000 bootstrap samples when the variable’s coefficient was not set to 0, i.e. the number of times the procedure determined the variable had a meaningful effect on outcome (see supplementary Tables 5 & supplementary Fig. 1). For the subset of biomarkers selected via elastic net, we refit ordinary logistic regression models to obtain unpenalized parameter estimates. A complete-case-per-timepoint approach was used. Unpenalized regression coefficients were standardized for comparability between biomarkers. Unpenalized models were internally validated using optimism correction on 1000 bootstrapped resamples, as described in Steyerberg et al. [39].

The ability of each biomarker – at the four timepoints – to discriminate patients with intracranial findings on CT or MRI from those without, was assessed with receiver operating curves (ROCs) and area under the curves (AUCs). The optimal pair of sensitivity and specificity was defined as the one corresponding to the Youden’s J statistic [40]. The corresponding specificities and thresholds with sensitivities set to 1 are also reported. Optimism-corrected AUCs, sensitivities and specificities are presented for the unpenalized multivariable models, with variables selected via elastic net. AUCs were interpreted using the following system: 0.90-1.00 = excellent, 0.80–0.90 = very good, 0.70–0.80 = moderate, 0.60–0.70 = poor, < 0.60 = negligible [41].

To provide some protection against false positives due to multiple comparisons, the significance level was set to α = 0.01. P-values for unpenalized regression models are not provided, as p-values after variable selection tend to be underestimated. A small number of blood biomarker outliers (*n* = 5) were determined and removed based on a pragmatic assessment of leverage values from LMMs and visual inspection of the data.

Statistical analyses were performed using R version 4.2.2 [42]. Linear mixed models were generated using the *nlme* package [43]. Post-hoc linear mixed model contrasts were conducted using the *emmeans* package [44]. Elastic net regression was conducted using the *glmnet* package [45]. Unpenalized logistic regressions were conducted in base R. AUC and ROC curves were computed using the *pROC* package [46]. Optimism correction was performed based on code adapted from Alberti [47].

## Results

**Fig. 1:**
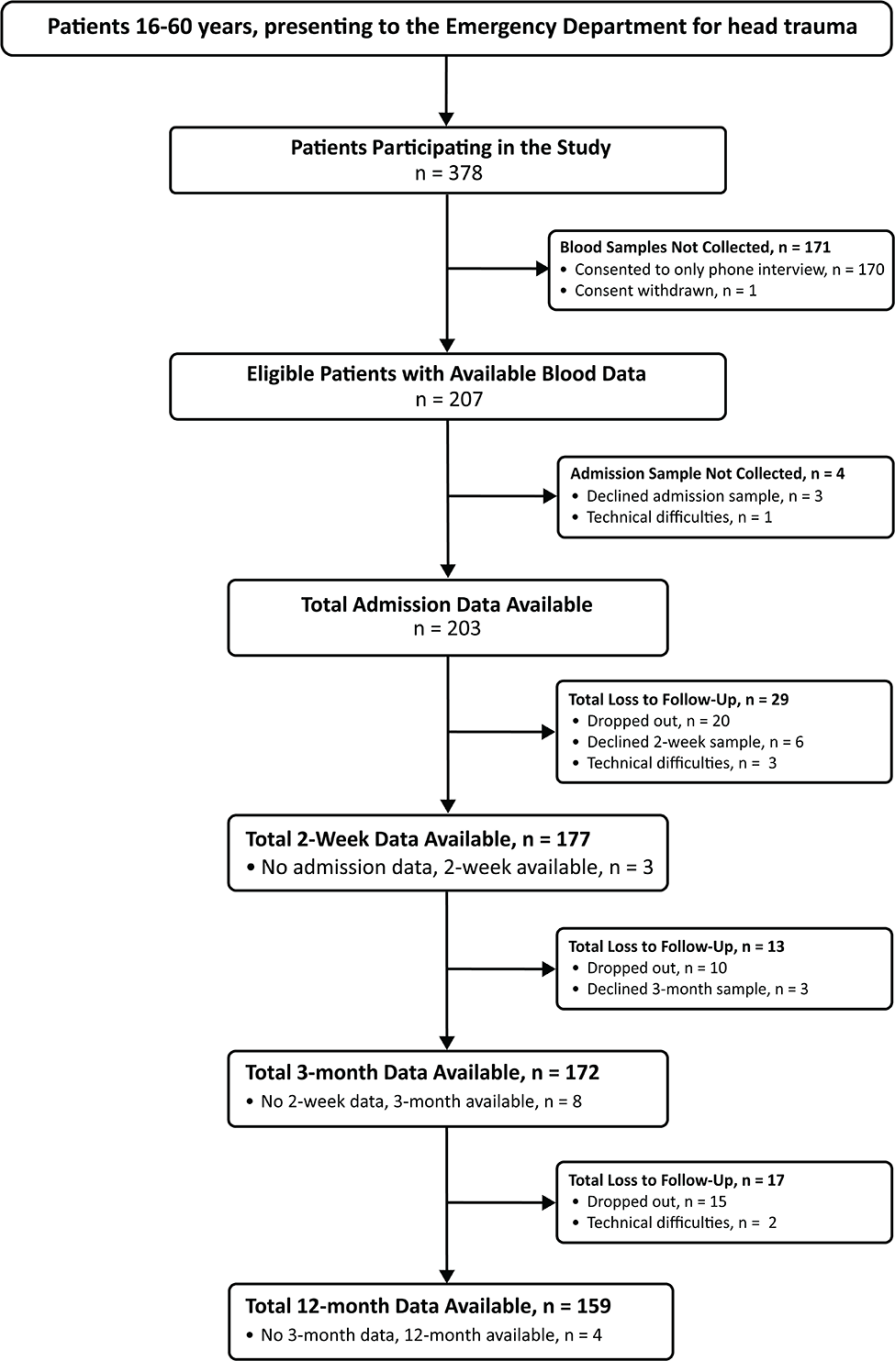
Flow chart depicting enrolment and follow-up of patients with mTBI from admission to 12 months. mTBI, mild traumatic brain injury

The flow chart in Fig. 1 summarizes sample numbers for each timepoint and reasons for drop out/data loss. 207 had blood data at one or more timepoints. At 2 weeks there were 177 with blood data available, at 3 months 172, and by 12 months, 159 patients remained in the study, giving a long-term retention rate of 77%.

Table 1 provides a detailed summary of the demographic and clinical characteristics of the patients with mTBI included. Most were men (63.3%), with GCS scores of 15 in 76.3%. LOC was observed in 47.3%, and PTA between 1 h and 24 h in 30.9%, while 36.7% experienced concurrent extracranial injuries. 16% were not triaged to CT and therefore excluded from analyses related to CT+/CT-. A total of 8% of patients were CT + and 12% were MRI+. 40.0% of patients had the same intracranial injuries on CT and MRI, 24.0% had additional or different findings on MRI compared to CT and 36.0% had findings on MRI but none on CT. In the CT+/MRI + groups specifically, loss-to-follow up led to *n =* 10 for CT + patients at 2 weeks, 3 months and 12 months, and for MRI + patients: *n =* 18 at 2 weeks and 12 months, and *n* = 19 at 3 months.

**Table 1:** Demographic and injury characteristics of total patients with mild TBI included in the study

	Patients with mTBI
	*N* = 207
**Sex (%)**	
Male	131 (63.3)
Females	76 (36.7)
**Age at Injury**	
Mean age, y (SD)	32.4 (13.2)
Age range, y	16–60
**GCS score (%)**	
13	5 (2.4)
14	33 (16.0)
15	158 (76.3)
Not recorded	11 (5.3)
**LOC (%)**	
Unobserved LOC	109 (52.7)
Observed LOC	98 (47.3)
**PTA duration (%)**	
PTA < 1 h	143 (69.1)
PTA between 1–24 h	64 (30.9)
**Injury Mechanism mTBI (%)**	
Fall	79 (38.1)
Traffic Accident	57 (27.5)
Sports Accident	26 (12.6)
Violence	31 (15.0)
Hit Object & Other	14 (6.8)
**Extracranial Injuries** ^‡^	
No	131 (63.3)
Yes	76 (36.7)
**Intracranial Finding on CT (%)**	
Contusion only	4 (1.9)
Intracranial hematoma only*	9 (4.4)
Contusion and hematoma*	3 (1.4)
No findings	158 (76.3)
Not triaged to CT	33 (16.0)
**Intracranial Finding on MRI (%)**	
TAI only	6 (2.9)
Contusion only	3 (1.4)
Intracranial hematoma only*	5 (1.4)
TAI and contusion	5 (2.4)
Contusion and hematoma*	6 (2.9)
No findings	182 (88.0)
**Intracranial Finding on CT vs. MRI (%)**	
Contusion on CT and MRI	2 (1.0)
Intracranial hematoma* on CT and MRI	5 (2.4)
Contusion and hematoma* on CT and MRI	3 (1.4)
Contusion on CT and contusion and TAI on MRI	2 (1.0)
Hematoma* on CT and contusion and hematoma* on MRI	3 (1.4)
Hematoma* on CT and contusion on MRI*	1 (0.5)
TAI and contusion on MRI, no CT findings	3 (1.4)
TAI findings on MRI, no CT findings	5 (2.4)
TAI findings on MRI, CT not performed	1 (0.5)
No findings on either modality incl. not triaged to CT	182 (88.0)

*Abbreviations*: mTBI = mild traumatic brain injury; GCS = Glasgow Coma Score; LOC = Loss of Consciousness; PTA = Post-Traumatic Amnesia; MRI = Magnetic Resonance Imaging

^‡^ Extracranial injuries refer to the presence of concurrent injuries to parts of the body other than the head (e.g. bone fracture). * Intracranial hematoma includes epidural hematomas, subdural hematomas, and traumatic subarachnoid hemorrhaging

### Longitudinal evolution of biomarkers based on presence of intracranial findings

**Fig. 2:**
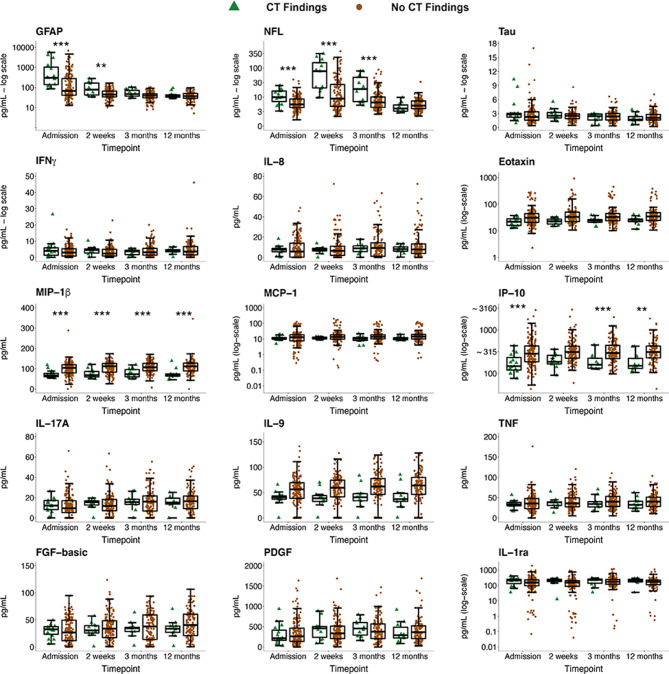
Biomarker concentrations over time in CT + and CT- patients with mTBI. Biomarker concentrations are presented as box plots with median as the midline, box borders representing the 25th and 75th percentile and whiskers calculated as the 25th and 75th percentile + 1.5 * interquartile range. Points above and below the whiskers represent outliers. Individual data points are presented within the boxplots. ** = *p* < 0.01; *** = *p* < 0.001. GFAP = Glial Fibrillary Acidic Protein; NFL = Neurofilament Light; Tau; IFNγ = Interferon Gamma; IL-8 = Interleukin-8; Eotaxin; MIP-1β = Macrophage Inflammatory Protein-1β; MCP-1 = Monocyte Chemoattractant Protein-1; IP-10 = IFNγ-induced Protein-10; IL-17A = Interleukin-17A; IL-9 = Interleukin-9; TNF = Tumor Necrotic Factor; FGF-basic = Basic Fibroblast Growth Factor; PDGF = Platelet-derived Growth Factor IL-1ra = Interleukin-1ra

**Fig. 3:**
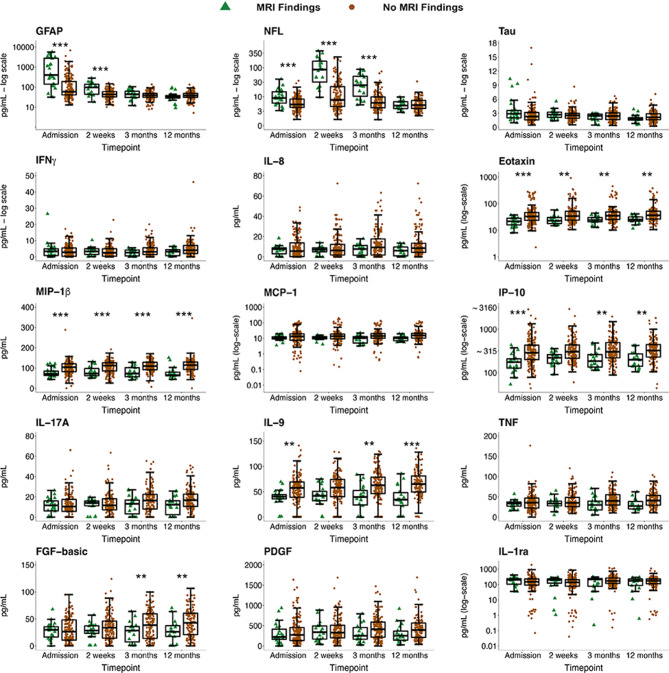
Biomarker concentrations over time in MRI + and MRI- patients with mTBI. Biomarker concentrations are presented as box plots with median as the midline, box borders representing the 25th and 75th percentile and whiskers calculated as the 25th and 75th percentile + 1.5 * interquartile range. Points above and below the whiskers represent outliers. Individual data points are presented within the boxplots. ** = *p* < 0.01; *** = *p* < 0.001. GFAP = Glial Fibrillary Acidic Protein; NFL = Neurofilament Light; Tau; IFNγ = Interferon Gamma; IL-8 = Interleukin-8; Eotaxin; MIP-1β = Macrophage Inflammatory Protein-1β; MCP-1 = Monocyte Chemoattractant Protein-1; IP-10 = IFNγ-induced Protein-10; IL-17A = Interleukin-17A; IL-9 = Interleukin-9; TNF = Tumor Necrotic Factor; FGF-basic = Basic Fibroblast Growth Factor; PDGF = Platelet-derived Growth Factor IL-1ra = Interleukin-1ra

Figures 2 and 3 depict the temporal profiles of the biomarkers based on CT + versus CT- and MRI + versus MRI-, respectively. Main effects from the final models used are presented in supplementary Table 3, and main effects from the models with sex and sex by group interaction included as covariates of interest are presented in supplementary Table 4 (due to statistical non-significance, sex effects are excluded from further analyses).

GFAP concentrations were significantly elevated in both CT+ and MRI+ patients compared to CT- and MRI- at admission and 2 weeks, while NFL was significantly elevated at admission, 2 weeks and 3 months (see Table 2). MIP-1β was significantly lower in CT+ and MRI+ patients at all timepoints, while IP-10 was significantly lower in CT+ and MRI+ patients at admission, 3 months and 12 months. Biomarkers uniquely associated with MRI+ were eotaxin, IL-9 and FGF-basic. Eotaxin was significantly lower in the MRI+ group at all timepoints, IL-9 was significantly lower at admission, 3 months and 12 months, and FGF-basic was significantly lower at 3 months and 12 months only. No biomarker was uniquely associated with CT+.

**Table 2:** Group contrasts of blood biomarker concentrations per timepoint between patients who were CT+/CT- and MRI+/MRI-

	Admission Estimate [95% CI] p-value	2 weeks Estimate [95% CI] p-value	3 months Estimate [95% CI] p-value	12 months Estimate [95% CI] p-value
CT Imaging				
GFAP ^†^	0.60 [0.30 – 0.90] *p* < 0.001	0.27 [0.10 – 0.45] *p* = 0.002	0.17 [0.03 – 0.30] *p* = 0.014	0.08 [-0.07 – 0.23] *p* = 0.306
NFL ^†^	0.23 [0.03 – 0.44] *p* = 0.027	0.67 [0.45 – 0.90] *p* < 0.001	0.44 [0.23 – 0.66] *p* < 0.001	-0.07 [-0.29 – 0.14] *p* = 0.511
MIP-1β	-24.26 [-38.28 – -10.23] *p* < 0.001	-26.54 [-40.05 – -13.02] *p* < 0.001	-25.98 [-41.01 – -10.95] *p* < 0.001	-29.27 [-44.50 – -14.03] *p* < 0.001
IP-10 ^†^	-0.25 [-0.39 – -0.11] *p* < 0.001	-0.17 [-0.31 – -0.02] *p* = 0.025	-0.23 [-0.36 – -0.11] *p* < 0.001	-0.24 [-0.38 – -0.09] *p* = 0.002
MRI Imaging				
GFAP ^†^	0.79 [0.51 – 1.07] *p* < 0.001	0.25 [0.12 – 0.39] *p* < 0.001	0.06 [-0.04 – 0.16] *p* = 0.264	-0.04 [-0.16 – 0.07] *p* = 0.463
NFL ^†^	0.21 [0.09 – 0.33] *p* < 0.001	0.85 [0.65 – 1.05] *p* < 0.001	0.55 [0.39 – 0.72] *p* < 0.001	-0.03 [-0.13 – 0.08] *p* = 0.631
Eotaxin	-0.23 [-0.36 – -0.10] *p* < 0.001	-0.20 [-0.33 – -0.07] *p* = 0.002	-0.19 [-0.32 – -0.07] *p* = 0.002	-0.18 [-0.30 – -0.05] *p* = 0.006
MIP-1β	-21.97 [-33.06 – -10.88] *p* < 0.001	-21.66 [-32.64 – -10.68] *p* < 0.001	-28.64 [-40.17 – -17.12] *p* < 0.001	-30.33 [-43.22 – -17.44] *p* < 0.001
IP-10	-0.26 [-0.37 – -0.14] *p* < 0.001	-0.14 [-0.26 – -0.02] *p* = 0.021	-0.20 [-0.32 – -0.08] *p* = 0.001	-0.19 [-0.31 – -0.07] *p* = 0.003
IL-9	-16.13 [-28.03 – -4.24] *p* = 0.008	-14.28 [-25.86 – -2.70] *p* = 0.016	-20.11 [-31.99 – -8.22] *p* = 0.001	-21.15 [-33.29 – -9.008] *p* < 0.001
FGF-basic	-2.15 [-11.14 – 6.84] *p* = 0.638	-7.90 [-17.13 – 1.34] *p* = 0.093	-11.51 [-20.26 – -2.77] p = **0.010**	-11.92 [-20.73 – -3.11] *p* = 0.008

^†^ Indicates base-10 log transformed data. Significant p-values are bolded (α = 0.01, adjusted using Tukey’s HSD). Presented biomarkers are those that exhibited a significant group by time interaction or a significant main effect of group. Estimate refers to mean group differences as estimated by the mixed model; 95% CI is the 95% confidence interval of the estimated group difference

mTBI, mild traumatic brain injury; GFAP, Glial fibrillary acidic protein; NFL, Neurofilament light

Contrasts comparing biomarker levels over time in patients with mTBI (Table 3) revealed a large, significant decrease in GFAP across both subgroups between admission and 2 weeks. There was also a significant decrease in GFAP between 2 weeks and 3 months in MRI+, MRI- and CT- groups. The difference between admission and 12-month GFAP levels was large and significant in all subgroups. There was a significant increase in NFL concentrations from admission to 2 weeks in all subgroups, followed by a significant decrease in NFL concentrations from 2 weeks and 3 months and also from 3 months to 12 months in all subgroups. The difference between 2-week and 12-month NFL levels was large and significant in both subgroups. Though there was no significant increase in FGF-basic in the MRI- group between successive timepoints, a steadily growing difference at every timepoint between admission and 12 months is evident, culminating in a statistically significant increase in FGF-basic concentrations at 12 months compared to admission in the MRI- group. There are no differences in FGF-basic in the MRI+ group, nor based on CT findings.

**Table 3:** Differences in blood biomarker concentrations between timepoints in patients who were CT+/CT- and/or MRI+/MRI-.

	Admission – 2 weeks Estimate [95% CI] p-value	2 weeks – 3 months Estimate [95% CI] p-value	3 months – 12 months Estimate [95% CI] p-value	Admission – 12 months Estimate [95% CI] p-value	2 weeks – 12 months Estimate [95% CI] p-value
**CT Imaging**					
**GFAP** ^*†*^					
CT+ Findings	-0.71 [-1.10 – -0.31] *p* < 0.001	-0.20 [-0.38 – -0.02] *p* = 0.026	-0.10 [-0.23 – 0.03] *p* = 0.206	-1.003 [-1.38 – -0.02] *p* < 0.001	
CT- Findings	-0.38 [-0.50 – -0.27] *p* < 0.001	-0.09 [-0.13 – -0.05] *p* < 0.001	-0.01 [-0.05 – 0.02] *p* = 0.742	-0.49 [-0.60 – -0.37] *p* < 0.001	
**NFL** ^*†*^					
CT+ Findings	0.75 [0.46 – 1.04] *p* < 0.001	-0.46 [-0.68 – -0.24] *p* < 0.001	-0.67 [-0.86 – -0.48] *p* < 0.001		-1.13 [-1.46 – -0.79] *p* < 0.001
CT- Findings	0.31 [0.23 – 0.39] *p* < 0.001	-0.23 [-0.29 – -0.17] *p* < 0.001	-0.15 [-0.21 – -0.10] *p* < 0.001		-0.38 [-0.48 – -0.29] *p* < 0.001
**MRI Imaging**					
**GFAP** ^*†*^					
MRI+ Findings	-0.85 [-1.21 – -0.48] *p* < 0.001	-0.26 [-0.40 – -0.12] *p* < 0.001	-0.10 [-0.20 – -0.01] *p* = 0.018	-1.21 [-1.56 – -0.12] *p* < 0.001	
MRI- Findings	-0.31 [-0.40 – -0.22] *p* < 0.001	-0.06 [-0.09 – -0.03] *p* < 0.001	-0.006 [-0.04 – 0.02] *p* = 0.950	-0.38 [-0.47 – -0.29] *p* < 0.001	
**NFL** ^*†*^					
MRI+ Findings	0.89 [0.64 – 1.15] *p* < 0.001	-0.50 [-0.78 – -0.21] *p* < 0.001	-0.68 [-0.87 – -0.50] *p* < 0.001		-1.18 [-1.42 – -0.95] *p* < 0.001
MRI- Findings	0.25 [0.18 – 0.32] *p* < 0.001	-0.20 [-0.28 – -0.12] *p* < 0.001	-0.10 [-0.15 – -0.06] *p* < 0.001		-0.30 [-0.38 – -0.23] *p* < 0.001
**FGF-basic**					
MRI+ Findings	-1.88 [-9.25 – 5.49] *p* = 0.913	0.78 [-5.49 – 7.04] *p* = 0.989	1.71 [-2.49 – 5.91] *p* = 0.720	0.61 [-5.24 – 7.04] *p* = 0.993	
MRI- Findings	3.87 [-0.33 – 8.07] *p* = 0.084	4.39 [0.22 – 8.57] *p* = 0.035	2.12 [-2.63 – 6.86] *p* = 0.659	10.38 [5.59 – 15.17] *p* < 0.001	

^†^Indicates base-10 log transformed data. Significant p-values are bolded (α = 0.01, adjusted using Tukey’s HSD). Presented biomarkers are those that exhibited a significant group by time interaction. Estimate refers to mean timepoint differences as estimated by the mixed model; 95% CI is the 95% confidence interval of the estimated timepoint difference

mTBI, mild traumatic brain injury; GFAP, Glial fibrillary acidic protein; NFL, Neurofilament light

### Single and multi-biomarker panel discriminability for complicated mTBI based on CT & MRI

Supplementary Figs. 2 and 3 present the ROC curves discriminating CT+/MRI + vs. CT-/MRI- for individual biomarkers at each timepoint and supplementary Tables 6 and 7 provide the corresponding AUC values, sensitivities, and specificities, including specificities and thresholds with sensitivity maximized. Some notable single biomarkers for classifying CT+ were: admission GFAP (sensitivity = 1.00, specificity = 0.58, AUC = 0.78); 2-week NFL (sensitivity = 1.00, specificity = 0.54, AUC = 0.81); 2-week eotaxin (sensitivity = 1.00, specificity = 0.51, AUC = 0.76); MIP-1β at all timepoints (AUC = 0.79 at admission, 2 weeks and 3 months and AUC = 0.81 at 12 months). Notable biomarkers for discriminating MRI+ were: admission GFAP (sensitivity = 0.92, specificity = 0.63, AUC = 0.82); NFL at 2 weeks (sensitivity = 0.74, specificity = 0.90, AUC = 0.89) and 3 months (sensitivity = 0.68, specificity = 0.92, AUC = 0.86); and MIP-1β at 12 months (AUC = 0.81).

Table 4 presents the unpenalized odds ratios of the biomarkers selected by elastic net as important predictors of CT+ and MRI+. The algorithm determined a combination of GFAP, NFL, MIP-1β, IP-10 and eotaxin to be predictive of both CT+ and MRI+ at admission and 2 weeks, while IL-1ra was uniquely predictive of intracranial findings on CT at those timepoints. At 3 months, NFL, MIP-1β and IP-10 were selected as predictors for CT+ and MRI+. At 12 months, MIP-1β was predictive of findings in both modalities, while IP-10 uniquely predicted CT+, and IL-9 uniquely predicted MRI+. GFAP and NFL were positively predictive of intracranial findings (i.e. elevated in CT+/MRI + groups) while for all inflammation markers, except IL-1ra, associations were negative (significantly lower concentrations in CT+/MRI + groups).

**Table 4:** Unpenalized odds ratios of the algorithmically selected blood biomarkers predicting patients who were CT + and/or MRI+.

	Admission	2 weeks	3 months	12 months
	Odds Ratio [95% CI]	Odds Ratio [95% CI]	Odds Ratio [95% CI]	Odds Ratio [95% CI]
**CT Imaging**				
GFAP †	1.26 [0.62–2.60]	1.10 [0.41–3.22]		
NFL †	1.82 [0.91–3.84]	1.81 [0.69–4.81]	1.77 [1.02–3.20]	
MIP-1β	0.46 [0.21–0.94]	0.45 [0.17–1.15]	0.56 [0.25–1.21]	0.42 [0.13–1.20]
IP-10 †	0.63 [0.29–1.26]	0.66 [0.23–1.60]	0.43 [0.14–1.14]	0.57 [0.19–1.45]
Eotaxin †	0.64 [0.26–1.21]	0.54 [0.14–1.09]		
IL-1ra	2.30 [1.07–6.68]	2.04 [0.73–12.71]		
**MRI Imaging**				
GFAP †	2.59 [1.44–4.93]	1.02 [0.45–2.23]		
NFL †	1.47 [0.82–2.71]	4.52 [2.14–11.13]	3.37 [1.99–6.30]	
MIP-1β	0.56 [0.29–1.07]	0.78 [0.38–1.68]	0.49 [0.25–0.90]	0.36 [0.13–0.88]
IP-10 †	0.57 [0.29–1.05]	0.45 [0.16–1.10]	0.55 [0.25–1.14]	
Eotaxin †	0.77 [0.40–1.27]	0.44 [0.14–0.87]		
IL-9				0.62 [0.28–1.36]

^†^Indicates base-10 log transformed data. The blood biomarkers were algorithmically selected via elastic net. Each marker of inflammation or CNS injury was included in the model as a possible predictor, at their respective timepoints. The biomarkers selected for each model were first standardized for comparability, before being input into an ordinary logistic regression, from which the above odds ratios, with 95% CIs (confidence intervals) were calculated. 95% CI = 95% confidence interval; GFAP = Glial fibrillary acidic protein; NFL = neurofilament light; MIP = Macrophage Inflammatory Protein; IP = IFNγ-induced Protein; IL = Interleukin

**Fig. 4:**
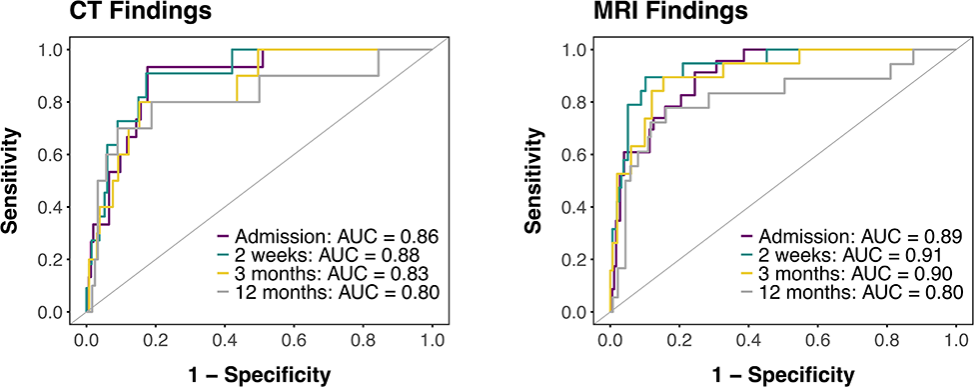
ROC curves indicating diagnostic accuracy for the algorithmically selected biomarker combinations discriminating CT + and MRI + patients. ROC curves are based on the optimal combination of biomarkers for predicting CT + and MRI + patients at each timepoint. AUC values for each timepoint are indicated in the plot. Biomarkers included for findings on CT: **Admission**: GFAp, NFL, MIP-1β, IP-10, eotaxin, IL-ra; **2 weeks**: GFAp, NFL, MIP-1β, IP-10, eotaxin, IL-ra **3 months**: NFL, MIP-1β, IP-10. **12 months**: MIP-1β, IP-10. Biomarkers included for findings on MRI: **Admission**: GFAp, NFL, MIP-1β, IP-10, eotaxin; **2 weeks**: GFAp, NFL, MIP-1β, IP-10, eotaxin **3 months**: NFL, MIP-1β, IP-10. **12 months**: MIP-1β, IL-9. ROC, Receiver Operating Characteristic; AUC, Area Under the Curve; GFAP = Glial fibrillary acidic protein; NFL = Neurofilament light; IFNγ = Interferon Gamma; IL = Interleukin; MIP = Macrophage Inflammatory Protein; MCP = Monocyte Chemoattractant Protein; IP = IFNγ-induced Protein; TNF = Tumor Necrotic Factor; FGF-basic = Basic Fibroblast Growth Factor; PDGF = Platelet-derived Growth Factor

Figure 4 illustrates the ROC curves of the selected combination of biomarkers at each timepoint for classifying CT+/MRI + and optimism-corrected AUC values are presented in Table 5. The multivariable predictions yielded AUCs above 0.80 at all timepoints for both modalities, with AUCs > 0.85 for discriminating CT+ from CT- at 2 weeks, and AUC ≈ 0.90 for discriminating MRI+ from MRI- at admission, 2 weeks and 3 months.

**Table 5:** Classification accuracy of unpenalized models with algorithmically-selected biomarkers for discriminating patients who were CT + and/or MRI+

	Sensitivity	Specificity	AUC [95% CI]	Included Biomarkers
CT Imaging				
Admission	0.90	0.77	0.86 [0.76–0.94]	GFAp, NFL, MIP-1β, IP-10, Eotaxin, IL-ra
2 weeks	0.94	0.68	0.88 [0.71–0.96]	GFAp, NFL, MIP-1β, IP-10, Eotaxin, IL-ra
3 months	0.72	0.85	0.83 [0.71–0.94]	NFL, MIP-1β, IP-10
12 months	0.76	0.80	0.80 [0.66–0.99]	MIP-1β, IP-10
MRI Imaging				
Admission	0.90	0.71	0.89 [0.84–0.95]	GFAp, NFL, MIP-1β, IP-10, Eotaxin
2 weeks	0.86	0.86	0.91 [0.85–0.98]	GFAp, NFL, MIP-1β, IP-10, Eotaxin
3 months	0.87	0.83	0.90 [0.84–0.97]	NFL, MIP-1β, IP-10
12 months	0.76	0.82	0.80 [0.69–0.93]	MIP-1β, IL-9

Optimism-corrected area under the curve (AUCs), sensitivities and specificities from unpenalized models with the selected combination of blood biomarkers – selected via elastic net – for discriminating patients who were CT+ and/or MRI+. Biomarker coefficients set to 0 in all models: Tau, IFNγ, IL-8, MCP-1, IL-17 A, TNF, FGF-basic, PDGF.

mTBI, mild traumatic brain injury; ROC, Receiver Operating Characteristic; AUC, Area Under the Curve; GFAP = Glial fibrillary acidic protein; NFL = Neurofilament light; IFNγ = Interferon Gamma; IL = Interleukin; MIP = Macrophage Inflammatory Protein; MCP = Monocyte Chemoattractant Protein; IP = IFNγ-induced Protein; TNF = Tumor Necrotic Factor; FGF-basic = Basic Fibroblast Growth Factor; PDGF = Platelet-derived Growth Factor

## Discussion

In this longitudinal study based on a representative sample of mixed-mechanism mild TBI patients with follow-up over one year, we present novel findings regarding the diagnostic utility and differential temporal dynamics of several blood-based biomarkers with regard to intracranial injuries determined on acute-phase CT and MRI. We evidenced the diagnostic utility of two blood-based biomarkers of CNS injury for discriminating complicated mTBI that differed based on timepoint of sampling after injury, representing a novel finding. Additionally, we evidenced heretofore undiscovered inflammation suppression in those with complicated mTBI that persisted over the entire year of follow-up after injury, along with 3 inflammation markers uniquely associated with traumatic intracranial findings on MRI, as opposed to CT.

### Biomarkers of CNS injury demonstrating high diagnostic utility acute, subacute and late phases for acute intracranial findings

The CNS-injury markers NFL and GFAP were highly discriminative biomarkers for both CT+ and MRI+. We present novel findings regarding the ability of NFL measured at 2 weeks and 3 months to classify patients with mTBI who were CT+ or MRI+, given previous studies [20–22] did not study NFL sampled at these timepoints. Single-biomarker classification accuracy of CT+/MRI + is highest using NFL sampled at 2 weeks, while 3-month NFL also demonstrates high discriminability, though with the optimal threshold yielding higher specificity (0.87) than sensitivity (0.60). Based on our results, we recommend NFL as a surrogate diagnostic biomarker of intracranial injury for patients who present to the clinic after 2 weeks especially, and potentially for up to 3 months after injury.

GFAP’s diagnostic utility at admission is in line with previous research and current clinical recommendations [10]. GFAP in our sample demonstrated perfect sensitivity for discriminating CT+ patients at admission and high sensitivity for discriminating MRI+ patients, adding to the already solid evidence for its early diagnostic utility across imaging modalities [10, 21, 22, 48, 49]. Additionally, we demonstrated good diagnostic accuracy of GFAP sampled at 2 weeks with the optimal threshold yielding a high specificity (0.99), but low sensitivity (0.36). This constitutes a new finding that could guide future studies and clinical recommendations.

### Evidence of persistent immunosuppression in individuals with complicated mTBI

In this study, we demonstrated primarily negative associations of several inflammation markers (see Figs. 2 and 3) with traumatic intracranial findings on both CT and MRI, which persisted for the whole year of follow-up, thereby reflecting chronic inflammation suppression in those with complicated mTBI. Although our findings regarding classification accuracy do not suggest that single inflammation markers could be useful diagnostically, these findings are nevertheless important from a biological and pathomechanistic perspective.

It is difficult to conceptualize why inflammation might be suppressed in those with a purportedly more serious injury, though there is precedence for this in the literature. In a severe TBI cohort, Kumar et al. (2015) [50] showed that those with concurrent polytrauma exhibited significantly lower circulating IL-6 levels at sub-acute and chronic phases than those with isolated TBI, despite initially higher acute levels. In our study, we demonstrated significantly lower levels of several inflammatory markers already during the acute stage that persist over time, a profile which differs from that generally found in moderate-severe TBI cohorts [24, 25, 50]. Similarly, our results mirror those from a seminal study published by Berger et al. (2009) [51], where researchers assessed the utility of a large multiplex panel of 44 serum biomarkers to screen for mild TBI in infants suffering a head trauma due to child abuse. In line with our findings, researchers reported reductions in 5 inflammation markers, including eotaxin, in contrast with elevations for other traditional inflammatory biomarkers, such as IL-6, and structural biomarkers such as neuron-specific enolase (NSE).

Additionally, it is well known there is little to no correlation between inflammatory markers in blood compared to cerebrospinal fluid [52], indicating there are likely different inflammatory mechanisms underlying central and peripheral inflammation. Unfortunately, it is impossible to tease apart contributions from neural sources due to breakdown in the blood-brain barrier (loss of blood brain barrier integrity), activation of immune cells in the glymphatic system, and inflammatory responses arising from peripheral sources for the inflammatory response observed here. Nevertheless, our results do point to any intracranial injury as a result of mTBI leading to a differential inflammatory response compared to mTBI without visible pathology on neuroimaging.

Moreover, despite the group differences found in this study, the absolute values of circulating inflammation markers are far lower than those found in severe TBI cohorts. The clinical consequence of the differences in immunoresponses in complicated versus uncomplicated mTBI is therefore unclear. In our recently published paper using the same sample as here [35], we demonstrated modest associations between inflammation factors and persisting post-concussion symptoms (PPCS), including both positive and negative effects of inflammation biomarkers that differed based on sampling time-point after mTBI. PPCS was associated predominantly with acute inflammatory processes, rather than ongoing inflammation or CNS-injury biomarkers, indicating a lack of association between ongoing peripheral inflammation and poor outcome. Therefore, the significance of the chronic immunosuppression demonstrated here calls for investigation and replication in future, better powered studies.

### Inflammation markers associated with traumatic intracranial findings on both CT & MRI

Specifically, the inflammation markers MIP-1β and IP-10 demonstrated significant reductions in both patients who were CT + and MRI + at almost all timepoints. Both markers are chemokines best known for their proinflammatory and chemotactic effects [53, 54]. MIP-1β is a key player in many inflammatory conditions, but also appears to be critical for wound healing and has the ability to promote homeostasis [53], while IP-10 plays an important role in CNS inflammation in a number of diseases, such as multiple sclerosis and Alzheimer’s disease [54, 55]. Studies have previously evidenced MIP-1β and IP-10 upregulation post-injury in both animal models of TBI [56–58] and human TBI studies [59–61]. We have also confirmed that both biomarkers are elevated compared to controls in a previous analysis on this sample [36]. However, despite studies reporting associations between poorer TBI outcome and both chemokines [62, 63], a recent study comparing the chronic phase of mTBI in rats and humans reported a positive correlation between MIP-1β and IP-10 concentrations and fractional anisotropy in several brain regions [59], interpreted as better white matter integrity as a function of higher concentrations of both chemokines. A similar relationship – higher levels of IP-10 and MIP-1β in those without intracranial findings – is reported here.

### Inflammation markers uniquely associated with traumatic intracranial findings on MRI

The growth factor FGF-basic, along with IL-9 and eotaxin were uniquely associated with MRI findings, perhaps due to biological mechanisms associated with TAI (TAI is the primary difference between MRI and CT groups, see Table 1). IL-9 and eotaxin were significantly reduced in individuals who were MRI+ (but not CT+) at almost all timepoints, while FGF-basic showed a steady increase in the MRI- group only, culminating in statistically significantly greater concentrations in those who were MRI- compared to MRI+ at 3 and 12 months.

FGF-basic is a fibroblast growth factor (FGF) believed to broadly promote angio- and neurogenesis, to reduce pathogenic disruption of the blood-brain barrier and to increase neuronal survival [64–66]. Following experimental TBI, it has been shown in human cell cultures to reduce apoptosis of human brain endothelial cells [64] and to upregulate neuronal survival in the adult hippocampus of a TBI mouse model [65], along with alleviating neurological deficits. Based on these findings, FGFs were recently proposed as a therapeutic treatment for stroke, which could have relevance also for patients with TBI [66]. Given FGFs’ evidenced neuroprotective effects, our results could indicate that MRI- patients (presumably, those without TAI) begin to naturally produce this beneficial growth factor given time, while the more severely injured MRI + patients are unable to do so within the first year following injury. Patients with mTBI who are MRI + may therefore represent a clinical target who would benefit especially from FGF therapies, though these results need confirmation in a larger sample.

Eotaxin is a chemokine that has long been associated with cognitive decline during aging in both humans and rodent models [67]. Its elevations have recently been detected in a number of neurodegenerative and psychiatric disorders [67], and it is particularly associated with memory deficits in Alzheimer’s disease [68]. In both animal models and human studies of more severe TBI, eotaxin has exhibited elevation in response to injury [62, 63, 69]. It is therefore unclear why no TAI would result in greater concentrations of eotaxin. We have previously shown that patients with mTBI without other injuries (e.g. skin contusions, abrasions, bone fractures etc.) also have greater eotaxin levels [36]. Taken together, eotaxin in our sample appears to be most elevated in those with the “mildest” form of mTBI, which is in line with the aforementioned study by Berger et al. (2009) [51]. These effects warrant replication and further investigation into their underlying mechanisms in both human and animal studies.

IL-9 is a pleiotropic cytokine primarily activated by Th9 cells [70]. Its major functions remain relatively underinvestigated, though it has been associated with a number of inflammatory diseases, specifically with regard to promoting immunotolerance [70, 71]. Though some rodent studies have evidenced IL-9 elevation following mTBI [72, 73], there is a dearth of research into its association with diagnostic and prognostic factors in human mTBI. Given IL-9 was algorithmically selected for the 12-month model predicting MRI findings, but not the corresponding CT model, it appears to demonstrate diagnostic specificity for MRI+ above and beyond other biomarkers in the late/chronic phase of mTBI. The results presented here, coupled with our previous findings of IL-9 elevations in patients with PPCS [35], warrant further investigation into the neurobiological mechanisms of both high and low IL-9 levels in mTBI.

### Multi-biomarker panels improve discriminability over single biomarkers at all timepoints

Single-biomarker discriminability for intracranial findings (CT+/MRI+) was poor to moderate for all inflammation markers and very good for GFAP sampled at admission and NFL sampled at 2 weeks and 3 months. Using a multi-biomarker panel with biomarkers selected via elastic net regression improved discriminability at all timepoints, with very good discriminability for CT+ at admission and 2 weeks (AUC > 0.85) and excellent discriminability for MRI+ at admission, 2 weeks and 3 months (AUC ≈ 0.90). Though IL-ra did not exhibit significant group differences in the LMMs, it was included in the model predicting CT+ status at admission and 2 weeks and was the only inflammation marker to show a positive association with intracranial findings. IL-ra is an endogenous receptor antagonist of IL-1ra [74] that is under investigation as a therapeutic target for TBI [75, 76]. Its elevation in CT+ could therefore reflect endogenous repair mechanisms, although given it was only included in some CT models (but not MRI models), the clinical/biological relevance of this effect remains unclear.

Put into context, our results show that a biomarker panel can identify with high accuracy patients with intracranial findings on CT/MRI, although similar discriminability can be achieved using only admission GFAP or 2-week NFL. Moreover, a greater number of biomarkers are discriminable of intracranial findings at early timepoints, though good discriminability is also achieved with a small selection of biomarkers at later timepoints. A validated panel of biomarkers for diagnosing intracranial injury late could help guide treatment plans in cases where CT/MRI were not performed acutely following injury. Taken together with our previous published works on this cohort (see [18, 35, 36]), we conclude that the biomarkers of CNS injury GFAP and NFL show high diagnostic utility for both intracranial findings on CT/MRI and for discriminating patients with mTBI from controls. Inflammation markers on the other hand show greater prognostic relevance for PPCS and remain elevated in patients with mTBI compared to controls for at least one year after injury. They showed diagnostic relevance also for intracranial findings on CT/MRI, but at levels lower than their CNS-injury counterparts. This is biologically unexpected and calls into question their clinical utility as a blood-based biomarker, given it is much more difficult to implement a biomarker cut-off that is below, rather than above a certain threshold.

## Limitations

Our study acknowledges several limitations. Firstly, the sample is comprised of those who were willing to participate in comprehensive data collection, meaning it may not be generalizable to all patients with mTBI. Furthermore, our upper-age limit of 60 years was designed to reduce the burden of age-related findings on MRI scans, however this means that known age-related effects of TBI were not investigated in this study. Furthermore, the small number of CT+/MRI+ cases compared to non-cases – which was further reduced due to drop-out – increases the likelihood of statistical overfitting and spurious results, as is reflected by relatively large confidence intervals and odds ratios that cross the threshold of 1. It is also prohibitive regarding investigation into any mediating factors underlying our determined effects of immunosuppression in those with complicated mTBI. For instance, effects of sex were investigated in this study and determined to be predominantly null, although it is difficult to know whether this would be true with greater statistical power, given there are well-known differences in inflammatory profiles between men and women [77]. We therefore do not present our models with the goal of generating accurate predictive models, but rather in the hopes that our findings can inform the selection of biomarkers for analysis in future, larger-scale studies, including outside of Norway (such as CENTER-TBI and TRACK-TBI). Nonetheless, we consider the small number of determined intracranial findings a consequence of recruiting a more representative sample from both the ED and ambulatory clinics.

Regarding biomarkers, we sampled total tau, although previous literature suggests phosphorylated tau, or the ratio of phosphorylated tau: total tau may be more relevant for TBI diagnostics [78, 79], and a new method for isolating brain-derived tau [80] may prove diagnostically superior in future studies. Similarly, IL-6 and IL-10 are two of the most studied inflammatory biomarkers in mTBI [81], however they were not expressed in sufficient quantities in our multiplex assay, de facto implying no effects of mTBI on their levels. A possible explanation for this is the higher expression of cytokines such as IL-6 in cerebrospinal fluid compared to blood [82]. Studies have also identified other potential diagnostic inflammation markers [83] that we unfortunately did not assess here. Lastly, due to technical constraints, our admission timepoint includes all blood drawn within 72 h from injury, although it is known that many biomarkers show greater discriminability within 24 h [18, 48, 81]. All-in-all, the limitations of our paper highlight the need for rigorous meta-analyses and pooling of data across labs to generate larger, and therefore more generalizable samples.

## Conclusions

This study provides novel evidence regarding the temporal dynamics and diagnostic utility of a large array of CNS-injury and inflammation biomarkers for identifying complicated mTBI in the acute to chronic phases after injury. We demonstrated NFL’s significant diagnostic utility at sub-acute (2 weeks) and potentially late (3 months) timepoints and confirmed GFAP’s acute diagnostic utility, along with evidencing its potential sub-acute diagnostic utility. We also shed light on interesting mechanisms of peripheral inflammation in complicated mTBI, whereby complicated mTBI appears to result in immunosuppression that is present from the acute phase and persists throughout the first year of injury, thus highlighting important biological differences between inflammatory profiles linked to traumatic intracranial findings in mild versus moderate-severe TBI. Using multi-biomarker panels improved diagnostic accuracy for traumatic intracranial findings at all timepoints, though our results suggest similar accuracy can be achieved using only acutely-sampled GFAP and NFL sampled at 2 weeks.

### Electronic supplementary material

Below is the link to the electronic supplementary material.

Supplementary Material 1

Supplementary Material 2

Supplementary Material 3

Supplementary Material 4

## Data Availability

Data, including biofluids, from the Trondheim mTBI study used in this manuscript can be accessed by contacting the last author (asta.haberg@ntnu.no) or Professor Toril Skandsen (toril.skandsen@ntnu.no) by e-mail. Note that data will only be shared with qualified investigators in connection with planned investigations which have undergone scientific and ethical review and are in compliance with the European Union General Data Protection Regulations (GDPR), Norwegian laws and regulations, and NTNU regulations. The completion of a material transfer agreement (MTA) signed by an institutional official will be required. Analytic code used to conduct the analyses presented in this study are not available in a public repository; they may be available by emailing the first author (gerard.clarke@ntnu.no).
