# Supplementary figures and images for: Chronic immunosuppression across 12 months and high ability of acute and subacute CNS-injury biomarker concentrations to identify individuals with complicated mTBI on acute CT and MRI
Source: J Neuroinflammation. 2024 Apr 27;21:109. doi: 10.1186/s12974-024-03094-8 (PMC11056044; doi:10.1186/s12974-024-03094-8)

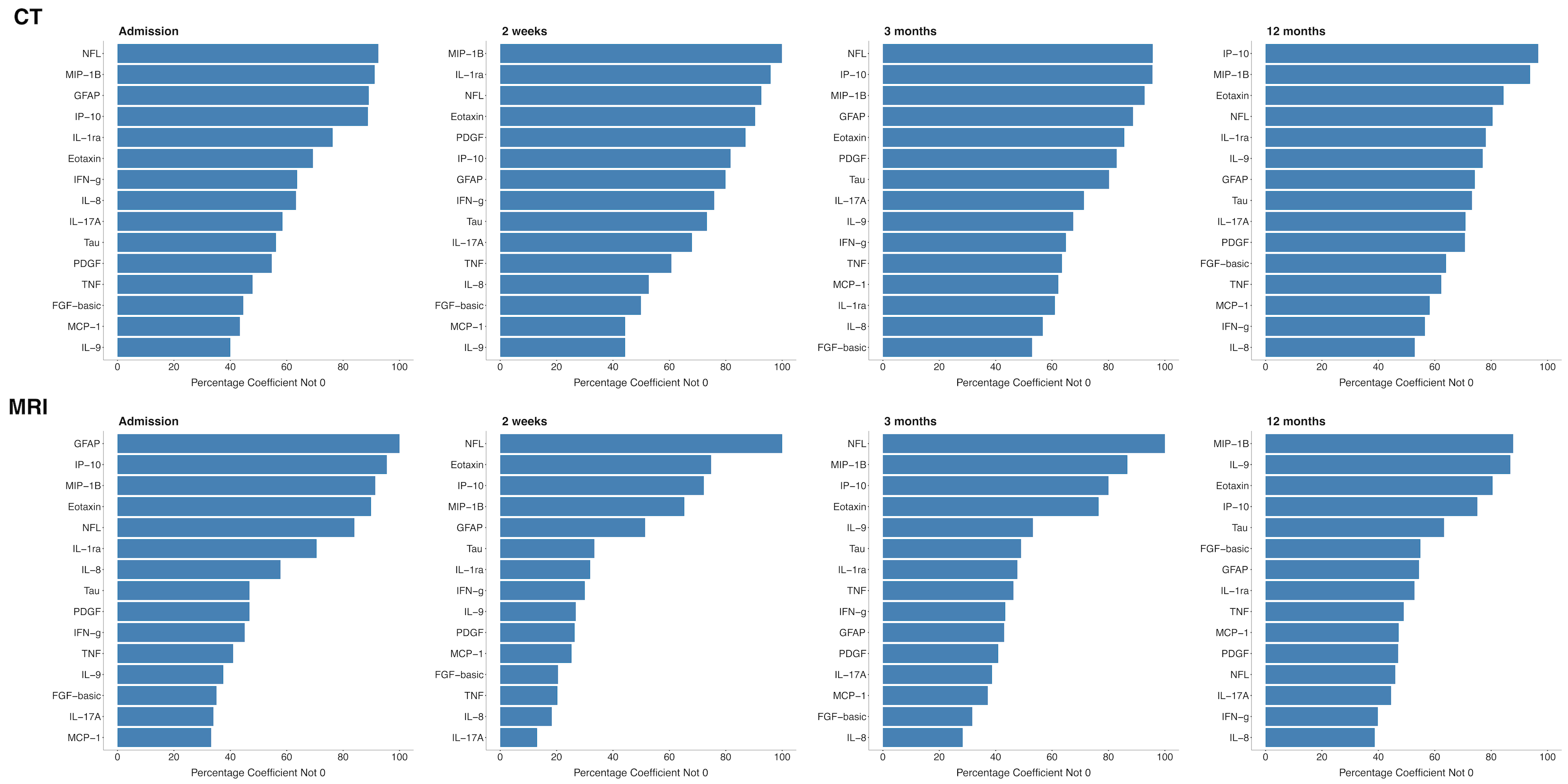

Supplement: Supplementary file 1 — Supplementary Material 1 [file 12974_2024_3094_MOESM1_ESM.tif]

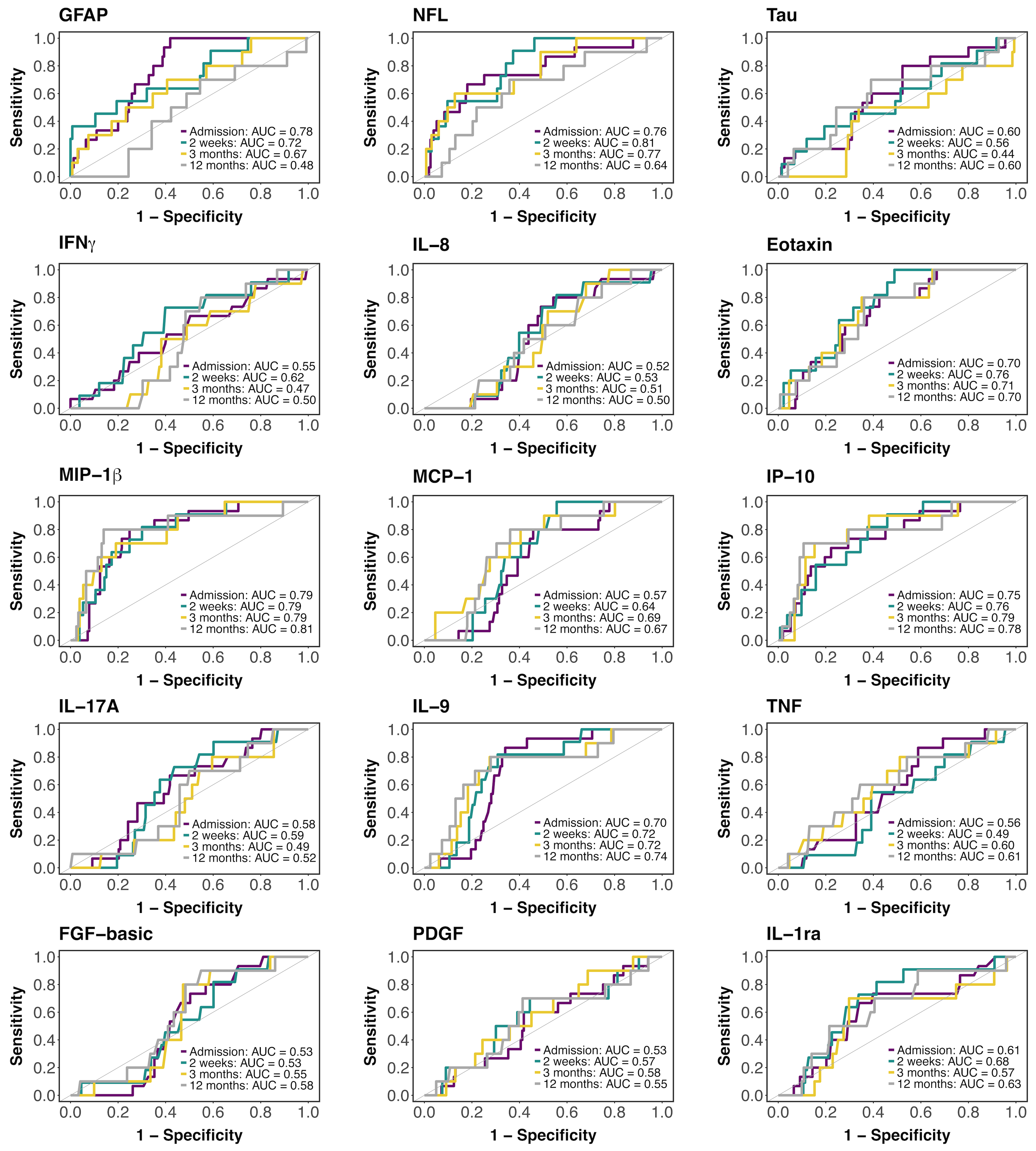

Supplement: Supplementary file 2 — Supplementary Material 2 [file 12974_2024_3094_MOESM2_ESM.tif]

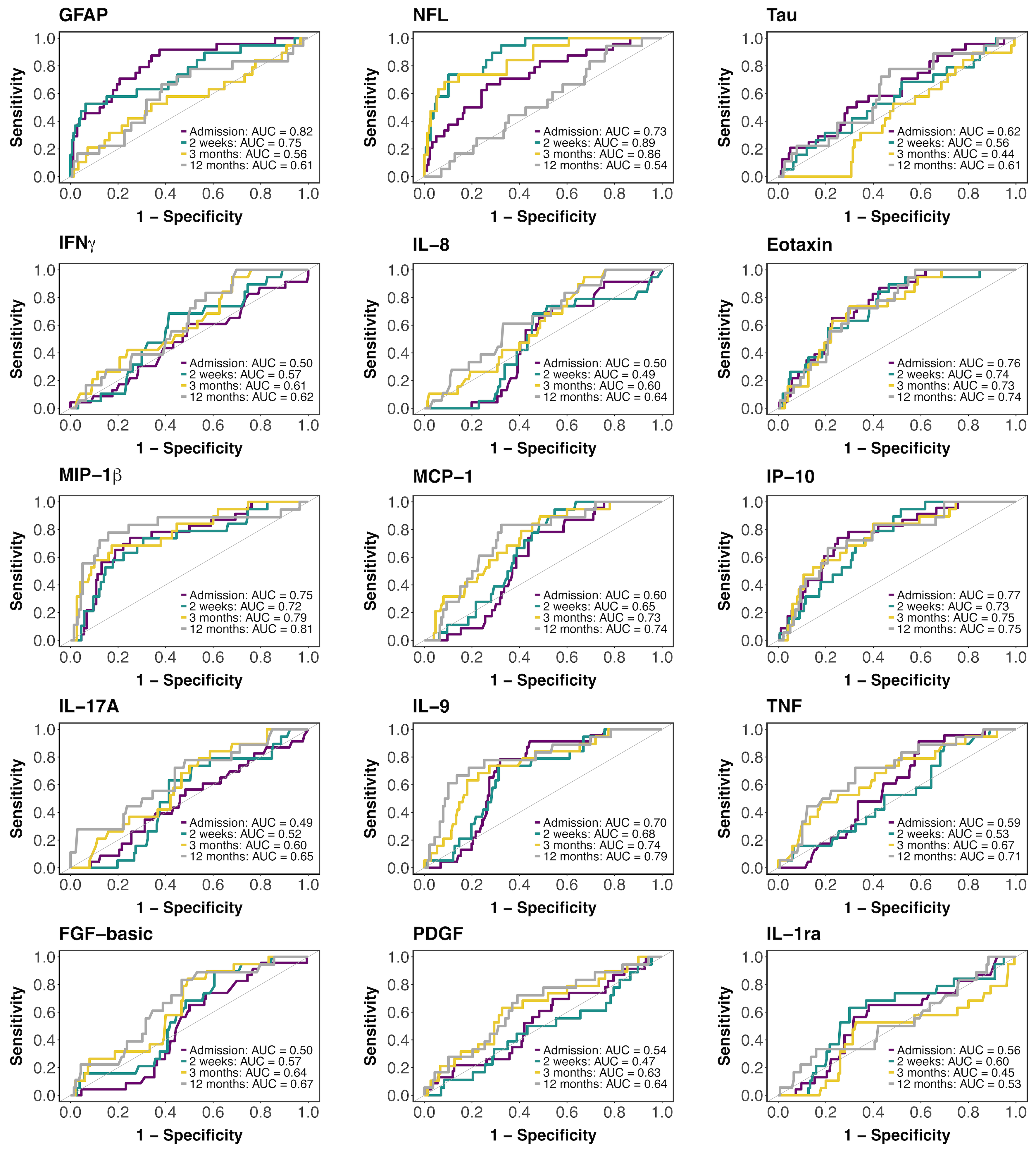

Supplement: Supplementary file 3 — Supplementary Material 3 [file 12974_2024_3094_MOESM3_ESM.tif]
