# Supplementary material for: Chronic immunosuppression across 12 months and high ability of acute and subacute CNS-injury biomarker concentrations to identify individuals with complicated mTBI on acute CT and MRI
Source: J Neuroinflammation. 2024 Apr 27;21:109. doi: 10.1186/s12974-024-03094-8 (PMC11056044; doi:10.1186/s12974-024-03094-8)
Supplement: Supplementary file 4 — Supplementary Material 4 [file 12974_2024_3094_MOESM4_ESM.docx]

**Supplementary Table 1:** Descriptive statistics of blood biomarkers at each timepoint, on their original scales, separated by patients with mTBI who were CT+ and CT-

|  | **Admission** | | **2 weeks** | | **3 months** | | **12 months** | |
| --- | --- | --- | --- | --- | --- | --- | --- | --- |
|  | **CT+** | **CT-** | **CT+** | **CT-** | **CT+** | **CT-** | **CT+** | **CT-** |
| **GFAP (pg/mL)** |  |  |  |  |  |  |  |  |
| Mean (SD) | 373.88 (862.82) | 1138.8 (1729.6) | 53.02 (30.35) | 108.76 (81.66) | 41.95 (19.92) | 56.57 (26.08) | 41.05 (20.02) | 44.78 (22.07) |
| Median (IQR) | 67.56 (240.06) | 302.74 (917.54) | 44.67 (33.24) | 77.72 (125.44) | 39.16 (17.24) | 46.82 (34.88) | 37.36 (21.00) | 35.79 (10.20) |
| Range | 12.92 - 6815.14 | 85.70 - 5634.25 | 10.20 - 165.35 | 33.85 - 281.45 | 7.82 - 106.12 | 29.30 - 104.54 | 5.19 - 96.77 | 28.65 - 94.11 |
| **NFL (pg/mL)** |  |  |  |  |  |  |  |  |
| Mean (SD) | 7.15 (5.18) | 11.88 (6.14) | 30.10 (53.88) | 102.95 (105.98) | 10.73 (13.26) | 31.00 (29.76) | 5.93 (3.51) | 4.73 (2.13) |
| Median (IQR) | 5.58 (4.22) | 9.91 (9.11) | 8.86 (22.50) | 78.40 (138.02) | 6.54 (5.67) | 19.18 (42.34) | 5.10 (3.37) | 4.03 (2.10) |
| Range | 1.64 - 40.34 | 3.30 - 25.05 | 2.16 - 379.24 | 9.59 - 315.45 | 2.58 - 88.53 | 5.27 - 79.96 | 2.20 - 33.68 | 2.89 - 9.76 |
| **Tau (pg/mL)** |  |  |  |  |  |  |  |  |
| Mean (SD) | 2.77 (2.08) | 3.50 (2.62) | 2.55 (1.06) | 2.83 (1.19) | 2.47 (1.13) | 2.10 (0.97) | 2.28 (1.11) | 1.97 (1.03) |
| Median (IQR) | 2.28 (1.80) | 2.82 (0.81) | 2.52 (1.04) | 2.54 (1.14) | 2.40 (1.39) | 2.45 (1.28) | 2.10 (1.18) | 1.74 (0.82) |
| Range | 0.44 - 16.97 | 0.95 - 10.31 | 0.51 - 8.66 | 1.37 - 5.54 | 0.38 - 6.65 | 0.45 - 2.96 | 0.22 - 7.11 | 0.64 - 3.83 |
| **IFNγ (pg/mL)** |  |  |  |  |  |  |  |  |
| Mean (SD) | 3.81 (3.50) | 5.10 (6.45) | 3.27 (3.31) | 4.16 (2.85) | 4.13 (3.91) | 3.18 (1.87) | 4.85 (5.25) | 3.96 (1.88) |
| Median (IQR) | 3.02 (4.53) | 4.05 (4.54) | 2.42 (4.12) | 4.51 (2.54) | 3.18 (4.20) | 3.76 (2.76) | 3.61 (5.13) | 4.00 (1.31) |
| Range | 0.01 - 17.21 | 0.03 - 26.50 | 0.01 - 22.79 | 0.11 - 10.37 | 0.01 - 20.09 | 0.05 - 5.65 | 0.02 - 46.12 | 0.21 - 6.44 |
| **IL-8 (pg/mL)** |  |  |  |  |  |  |  |  |
| Mean (SD) | 9.39 (10.57) | 7.28 (4.69) | 9.67 (11.36) | 7.28 (3.49) | 12.14 (13.28) | 8.55 (5.02) | 11.59 (12.80) | 8.05 (4.13) |
| Median (IQR) | 5.79 (13.72) | 7.75 (3.30) | 6.54 (9.12) | 7.75 (2.60) | 9.27 (13.32) | 8.99 (5.62) | 7.84 (9.39) | 8.30 (4.28) |
| Range | 0.01 - 48.87 | 0.05 - 18.42 | 0.01 - 72.37 | 0.05 - 13.92 | 0.01 - 63.29 | 0.22 - 17.86 | 0.01 - 72.37 | 0.25 - 13.57 |
| **Eotaxin (pg/mL)** |  |  |  |  |  |  |  |  |
| Mean (SD) | 46.65 (54.25) | 23.11 (8.26) | 54.76 (93.54) | 23.06 (6.34) | 49.22 (60.35) | 25.02 (8.22) | 50.77 (59.61) | 26.03 (8.59) |
| Median (IQR) | 31.10 (23.18) | 22.08 (12.21) | 33.03 (27.76) | 22.83 (9.44) | 32.48 (20.52) | 24.52 (5.50) | 34.53 (23.20) | 26.04 (5.70) |
| Range | 2.29 - 281.07 | 12.61 - 37.57 | 10.63 - 919.95 | 14.83 - 32.90 | 10.14 - 453.28 | 14.22 - 38.90 | 10.60 - 382.27 | 11.60 - 41.13 |
| **MIP-1β (pg/mL)** |  |  |  |  |  |  |  |  |
| Mean (SD) | 99.81 (33.58) | 73.25 (18.04) | 104.97 (29.11) | 75.16 (22.52) | 107.06 (28.30) | 77.97 (22.92) | 108.58 (35.49) | 75.28 (26.74) |
| Median (IQR) | 104.07 (40.46) | 66.81 (17.06) | 111.98 (42.74) | 68.01 (26.78) | 106.85 (36.11) | 72.52 (35.87) | 111.12 (38.75) | 68.44 (10.07) |
| Range | 0.17 - 288.21 | 52.89 - 117.61 | 25.99 - 173.46 | 50.19 - 121.19 | 7.05 - 170.55 | 49.94 - 118.74 | 0.37 - 345.47 | 45.65 - 138.38 |
| **MCP-1 (pg/mL)** |  |  |  |  |  |  |  |  |
| Mean (SD) | 19.45 (23.10) | 11.91 (4.03) | 21.76 (30.91) | 11.37 (1.93) | 20.01 (22.30) | 10.62 (5.25) | 20.68 (23.05) | 11.43 (3.70) |
| Median (IQR) | 13.04 (11.03) | 11.43 (2.92) | 13.61 (8.80) | 10.92 (3.12) | 13.96 (8.60) | 10.40 (4.23) | 14.38 (9.59) | 10.20 (3.23) |
| Range | 0.29 - 125.98 | 4.96 - 19.55 | 0.15 - 198.14 | 8.95 - 14.23 | 0.40 - 133.66 | 3.63 - 21.80 | 0.59 - 124.43 | 8.10 - 19.78 |
| **IP-10 (pg/mL)** |  |  |  |  |  |  |  |  |
| Mean (SD) | 386.43 (368.66) | 188.49 (102.60) | 393.03 (343.33) | 212.38 (77.39) | 378.55 (272.50) | 196.82 (100.22) | 374.69 (255.36) | 202.90 (119.73) |
| Median (IQR) | 284.14 (242.39) | 146.34 (115.36) | 311.18 (198.47) | 184.47 (94.64) | 293.64 (224.15) | 158.65 (92.55) | 309.58 (198.91) | 149.35 (94.33) |
| Range | 43.69 - 2868.15 | 76.51 - 433.21 | 60.10 - 2931.80 | 90.49 - 359.04 | 97.21 - 1963.44 | 125.48 - 450.61 | 43.99 - 1826.93 | 105.06 - 423.01 |
| **IL-17A (pg/mL)** |  |  |  |  |  |  |  |  |
| Mean (SD) | 11.76 (10.62) | 12.53 (7.09) | 13.35 (10.62) | 14.04 (5.30) | 15.80 (12.28) | 15.53 (7.95) | 16.45 (10.62) | 15.15 (6.90) |
| Median (IQR) | 9.56 (11.68) | 12.18 (8.92) | 11.87 (11.13) | 15.56 (4.11) | 16.07 (14.90) | 15.57 (5.07) | 16.48 (12.44) | 15.16 (5.90) |
| Range | 0.01 - 66.07 | 0.13 - 26.34 | 0.01 - 63.45 | 0.16 - 19.65 | 0.01 - 55.44 | 0.11 - 26.96 | 0.02 - 50.78 | 0.02 - 25.23 |
| **IL-9 (pg/mL)** |  |  |  |  |  |  |  |  |
| Mean (SD) | 51.74 (28.59) | 39.38 (13.44) | 56.25 (28.68) | 39.18 (18.47) | 60.46 (30.08) | 42.42 (23.48) | 62.46 (27.58) | 41.88 (24.54) |
| Median (IQR) | 56.31 (30.67) | 40.63 (6.49) | 59.66 (32.98) | 38.68 (11.62) | 61.95 (31.10) | 40.84 (13.69) | 64.05 (33.48) | 37.06 (15.58) |
| Range | 0.03 - 141.26 | 0.52 - 65.70 | 0.04 - 128.00 | 1.20 - 70.07 | 0.08 - 128.00 | 0.69 - 83.68 | 0.05 - 128.00 | 0.77 - 85.53 |
| **TNF (pg/mL)** |  |  |  |  |  |  |  |  |
| Mean (SD) | 37.80 (20.73) | 33.07 (10.53) | 40.23 (20.14) | 36.26 (12.94) | 42.69 (19.25) | 36.65 (16.91) | 41.99 (18.03) | 35.37 (14.30) |
| Median (IQR) | 35.64 (24.98) | 33.39 (8.30) | 36.12 (22.17) | 38.64 (8.75) | 40.15 (24.77) | 34.26 (12.66) | 40.15 (22.59) | 32.52 (16.78) |
| Range | 0.17 - 176.35 | 16.01 - 57.18 | 5.40 - 120.47 | 13.14 - 65.08 | 5.40 - 110.20 | 16.11 - 70.58 | 10.50 - 103.60 | 16.89 - 62.31 |
| **FGF-basic (pg/mL)** |  |  |  |  |  |  |  |  |
| Mean (SD) | 29.62 (22.56) | 29.63 (11.82) | 35.19 (23.89) | 31.86 (14.48) | 38.28 (25.74) | 33.02 (15.78) | 42.18 (25.70) | 33.63 (17.59) |
| Median (IQR) | 26.71 (38.02) | 32.59 (14.10) | 33.86 (31.11) | 30.55 (13.66) | 37.81 (45.40) | 34.59 (7.86) | 40.53 (39.79) | 33.59 (11.63) |
| Range | 0.07 - 94.91 | 5.49 - 49.23 | 0.38 - 123.92 | 0.93 - 57.17 | 0.20 - 93.80 | 2.64 - 63.93 | 0.32 - 106.59 | 1.80 - 70.25 |
| **PDGF** |  |  |  |  |  |  |  |  |
| Mean (SD) | 351.96 (308.12) | 305.73 (245.86) | 404.55 (287.07) | 443.87 (261.34) | 424.24 (276.00) | 463.34 (208.80) | 405.06 (275.29) | 365.03 (255.80) |
| Median (IQR) | 262.90 (327.08) | 214.96 (249.20) | 336.02 (303.81) | 457.98 (267.14) | 376.60 (359.98) | 454.60 (309.34) | 365.29 (312.74) | 288.07 (264.47) |
| Range | 0.17 - 1635.19 | 32.40 - 936.15 | 0.48 - 1684.96 | 88.66 - 883.52 | 0.17 - 1471.95 | 158.64 - 793.28 | 27.29 - 1689.88 | 85.34 - 917.77 |
| **IL-1ra (pg/mL)** |  |  |  |  |  |  |  |  |
| Mean (SD) | 189.76 (203.61) | 205.82 (102.05) | 170.74 (147.55) | 201.89 (78.11) | 215.92 (181.71) | 203.97 (95.23) | 190.35 (146.60) | 201.85 (76.56) |
| Median (IQR) | 153.96 (136.82) | 218.14 (110.56) | 153.96 (119.65) | 204.53 (71.08) | 176.02 (124.16) | 234.90 (112.44) | 170.25 (106.74) | 211.15 (80.93) |
| Range | 0.07 - 1913.50 | 35.63 - 412.66 | 0.04 - 926.01 | 12.90 - 291.26 | 0.16 - 1186.13 | 35.63 - 310.41 | 0.56 - 1101.81 | 35.63 - 296.54 |

GFAP = Glial Fibrillary Acidic Protein; NFL = Neurofilament Light; IFNγ = Interferon Gamma; IL = Interleukin; MIP = Macrophage Inflammatory Protein; MCP = Monocyte Chemoattractant Protein; IP = IFNγ-induced Protein; TNF = Tumor Necrotic Factor; FGF-basic = Basic Fibroblast Growth Factor; PDGF = Platelet-derived Growth Factor

**Supplementary Table 2:** Descriptive statistics of blood biomarkers at each timepoint, on their original scales, separated by patients with mTBI who were MRI+ and MRI-

|  | **Admission** | | **2 weeks** | | **3 months** | | **12 months** | |
| --- | --- | --- | --- | --- | --- | --- | --- | --- |
|  | **MRI+** | **MRI-** | **MRI+** | **MRI-** | **MRI+** | **MRI-** | **MRI+** | **MRI-** |
| **GFAP (pg/mL)** |  |  |  |  |  |  |  |  |
| Mean (SD) | 245.22 (640.03) | 1412.4 (1701.3) | 49.29 (26.67) | 101.19 (68.24) | 42.52 (20.64) | 48.56 (25.33) | 42.18 (20.52) | 38.24 (23.67) |
| Median (IQR) | 59.24 (149.97) | 405.65 (2590.61) | 43.14 (27.41) | 97.37 (94.87) | 39.16 (16.97) | 44.00 (32.18) | 38.64 (17.50) | 32.98 (9.66) |
| Range | 12.92 - 6815.14 | 31.62 - 5634.25 | 10.20 - 165.35 | 18.09 - 281.45 | 7.82 - 138.43 | 12.02 - 104.54 | 5.19 - 133.91 | 9.04 - 94.11 |
| **NFL (pg/mL)** |  |  |  |  |  |  |  |  |
| Mean (SD) | 6.80 (4.39) | 11.53 (8.20) | 21.20 (35.54) | 118.99 (108.52) | 8.71 (9.06) | 33.71 (27.22) | 6.00 (3.46) | 5.32 (1.88) |
| Median (IQR) | 5.38 (4.01) | 9.05 (8.80) | 7.74 (17.69) | 86.11 (136.73) | 6.17 (5.64) | 24.83 (40.28) | 5.13 (3.49) | 4.92 (2.81) |
| Range | 1.64 - 35.51 | 3.30 - 40.34 | 1.64 - 235.04 | 9.59 - 379.24 | 1.61 - 74.47 | 5.27 - 88.53 | 2.20 - 33.68 | 2.89 - 9.76 |
| **Tau (pg/mL)** |  |  |  |  |  |  |  |  |
| Mean (SD) | 2.65 (1.90) | 3.55 (2.52) | 2.55 (1.04) | 2.75 (1.01) | 2.50 (1.17) | 2.16 (0.76) | 2.28 (1.10) | 1.86 (0.87) |
| Median (IQR) | 2.28 (1.58) | 2.87 (1.57) | 2.51 (1.04) | 2.68 (1.09) | 2.40 (1.44) | 2.43 (1.08) | 2.10 (1.25) | 1.88 (0.51) |
| Range | 0.44 - 16.97 | 0.95 - 10.31 | 0.51 - 8.66 | 1.37 - 5.54 | 0.38 - 6.65 | 0.45 - 2.96 | 0.22 - 7.11 | 0.49 - 3.83 |
| **IFNγ (pg/mL)** |  |  |  |  |  |  |  |  |
| Mean (SD) | 3.71 (3.47) | 4.09 (5.45) | 3.22 (3.23) | 3.57 (2.52) | 4.07 (3.79) | 2.47 (1.96) | 4.94 (5.08) | 3.03 (2.23) |
| Median (IQR) | 2.83 (5.06) | 3.39 (4.13) | 2.48 (4.62) | 3.39 (3.52) | 3.09 (4.20) | 2.66 (3.66) | 4.05 (4.98) | 3.20 (3.69) |
| Range | 0.03 - 17.21 | 0.01 - 26.50 | 0.01 - 22.79 | 0.11 - 10.37 | 0.01 - 20.09 | 0.04 - 5.65 | 0.02 - 46.12 | 0.06 - 6.44 |
| **IL-8 (pg/mL)** |  |  |  |  |  |  |  |  |
| Mean (SD) | 9.35 (10.54) | 6.64 (4.56) | 9.67 (11.41) | 6.33 (3.88) | 12.52 (13.15) | 6.95 (5.42) | 12.25 (12.64) | 5.98 (4.68) |
| Median (IQR) | 5.69 (13.57) | 7.75 (5.83) | 6.27 (10.36) | 7.20 (3.89) | 9.32 (15.80) | 7.75 (9.60) | 8.59 (8.94) | 6.12 (8.74) |
| Range | 0.01 - 48.87 | 0.05 - 18.42 | 0.01 - 72.37 | 0.01 - 13.92 | 0.01 - 63.29 | 0.02 - 17.86 | 0.01 - 72.37 | 0.08 - 13.57 |
| **Eotaxin (pg/mL)** |  |  |  |  |  |  |  |  |
| Mean (SD) | 49.51 (54.31) | 22.05 (8.05) | 55.66 (88.34) | 25.31 (10.22) | 52.55 (61.37) | 25.73 (8.69) | 52.62 (56.84) | 26.55 (8.33) |
| Median (IQR) | 32.98 (23.11) | 21.71 (12.21) | 33.91 (27.66) | 22.83 (12.14) | 35.36 (23.11) | 24.34 (9.19) | 36.47 (25.75) | 25.41 (9.88) |
| Range | 2.29 - 281.07 | 7.99 - 37.57 | 10.63 - 919.95 | 14.83 - 58.00 | 10.14 - 453.28 | 12.91 - 44.26 | 10.60 - 382.27 | 11.60 - 41.13 |
| **MIP-1β (pg/mL)** |  |  |  |  |  |  |  |  |
| Mean (SD) | 101.01 (32.10) | 77.55 (22.62) | 105.46 (29.61) | 82.30 (26.76) | 109.13 (28.00) | 78.94 (26.29) | 110.76 (33.99) | 75.88 (28.76) |
| Median (IQR) | 103.80 (38.59) | 70.41 (21.41) | 111.98 (40.36) | 72.55 (34.87) | 109.40 (36.67) | 72.30 (45.07) | 114.22 (36.38) | 64.68 (17.31) |
| Range | 0.17 - 288.21 | 43.59 - 122.57 | 25.99 - 191.78 | 50.19 - 131.48 | 7.05 - 170.55 | 41.22 - 127.96 | 0.37 - 345.47 | 43.59 - 150.46 |
| **MCP-1 (pg/mL)** |  |  |  |  |  |  |  |  |
| Mean (SD) | 19.82 (23.38) | 11.35 (3.95) | 21.63 (29.96) | 10.78 (3.00) | 20.70 (22.57) | 9.77 (5.04) | 21.65 (22.96) | 10.45 (4.18) |
| Median (IQR) | 13.24 (11.87) | 11.02 (3.34) | 13.61 (9.56) | 10.92 (3.68) | 14.38 (9.93) | 10.20 (7.95) | 15.22 (10.62) | 9.99 (4.99) |
| Range | 0.08 - 125.98 | 3.54 - 19.55 | 0.15 - 198.14 | 4.16 - 16.36 | 0.40 - 133.66 | 3.26 - 21.80 | 0.59 - 124.43 | 5.62 - 19.78 |
| **IP-10 (pg/mL)** |  |  |  |  |  |  |  |  |
| Mean (SD) | 387.88 (349.30) | 188.50 (96.45) | 385.32 (327.46) | 218.09 (68.95) | 400.65 (298.09) | 218.33 (109.57) | 391.56 (272.36) | 220.44 (106.19) |
| Median (IQR) | 287.38 (231.85) | 175.14 (79.81) | 305.09 (222.41) | 220.37 (94.64) | 303.24 (272.89) | 184.78 (126.38) | 325.32 (215.10) | 198.50 (131.81) |
| Range | 43.69 - 2868.15 | 53.64 - 433.21 | 60.10 - 2931.80 | 90.49 - 359.04 | 88.57 - 1963.44 | 114.12 - 483.94 | 43.99 - 1826.93 | 105.06 - 423.29 |
| **IL-17A (pg/mL)** |  |  |  |  |  |  |  |  |
| Mean (SD) | 12.05 (10.39) | 10.88 (7.60) | 13.33 (10.64) | 11.82 (6.51) | 16.67 (12.91) | 11.63 (8.77) | 17.22 (10.66) | 11.53 (8.74) |
| Median (IQR) | 10.50 (11.84) | 11.57 (9.83) | 11.60 (10.97) | 13.95 (4.74) | 16.34 (14.50) | 13.15 (13.62) | 16.72 (12.18) | 12.36 (13.80) |
| Range | 0.01 - 66.07 | 0.01 - 26.34 | 0.01 - 63.45 | 0.07 - 19.65 | 0.01 - 55.44 | 0.08 - 26.96 | 0.04 - 50.78 | 0.02 - 25.74 |
| **IL-9 (pg/mL)** |  |  |  |  |  |  |  |  |
| Mean (SD) | 52.18 (29.40) | 38.28 (17.47) | 55.33 (29.44) | 40.14 (22.24) | 61.37 (30.58) | 37.87 (25.74) | 64.74 (27.61) | 35.65 (25.74) |
| Median (IQR) | 57.52 (30.15) | 40.63 (8.53) | 58.10 (34.12) | 41.93 (18.13) | 62.77 (32.20) | 39.76 (28.48) | 65.08 (31.80) | 34.37 (24.96) |
| Range | 0.03 - 141.26 | 0.52 - 69.50 | 0.04 - 128.99 | 0.20 - 75.13 | 0.17 - 129.98 | 0.08 - 83.68 | 0.05 - 135.94 | 0.61 - 85.53 |
| **TNF (pg/mL)** |  |  |  |  |  |  |  |  |
| Mean (SD) | 37.27 (20.33) | 31.58 (9.55) | 39.16 (20.25) | 33.93 (14.09) | 43.08 (19.07) | 31.90 (16.70) | 43.18 (19.00) | 30.42 (13.70) |
| Median (IQR) | 35.64 (24.14) | 33.39 (10.32) | 35.13 (22.76) | 33.39 (11.59) | 40.15 (25.29) | 29.90 (21.75) | 40.90 (23.87) | 27.30 (18.25) |
| Range | 0.17 - 176.35 | 16.01 - 57.18 | 8.97 - 120.47 | 5.40 - 65.08 | 10.37 - 110.20 | 5.40 - 70.58 | 10.50 - 103.60 | 10.75 - 62.31 |
| **FGF-basic (pg/mL)** |  |  |  |  |  |  |  |  |
| Mean (SD) | 28.87 (22.00) | 27.91 (15.15) | 33.95 (23.42) | 27.44 (14.82) | 38.91 (25.86) | 26.12 (18.75) | 42.85 (25.69) | 27.30 (19.91) |
| Median (IQR) | 26.44 (37.39) | 29.95 (19.03) | 33.86 (31.45) | 28.44 (13.73) | 38.45 (45.53) | 28.44 (28.46) | 42.70 (39.84) | 26.28 (19.84) |
| Range | 0.07 - 94.91 | 0.09 - 67.76 | 0.02 - 123.92 | 0.81 - 57.17 | 0.20 - 99.79 | 0.50 - 63.93 | 0.05 - 106.59 | 0.39 - 70.25 |
| **PDGF** |  |  |  |  |  |  |  |  |
| Mean (SD) | 354.60 (306.54) | 301.10 (250.29) | 396.60 (282.67) | 361.92 (242.19) | 444.24 (282.01) | 317.92 (226.15) | 423.73 (276.11) | 300.81 (223.06) |
| Median (IQR) | 272.80 (320.63) | 221.72 (249.20) | 328.61 (307.84) | 339.10 (321.94) | 412.84 (370.53) | 252.78 (287.02) | 394.79 (351.08) | 255.21 (225.73) |
| Range | 0.17 - 1635.19 | 0.31 - 936.15 | 0.48 - 1684.96 | 38.89 - 883.52 | 0.17 - 1471.95 | 24.95 - 793.28 | 40.74 - 1689.88 | 27.29 - 917.77 |
| **IL-1ra (pg/mL)** |  |  |  |  |  |  |  |  |
| Mean (SD) | 187.52 (196.40) | 184.43 (97.24) | 169.58 (150.16) | 173.61 (92.30) | 214.88 (176.76) | 165.69 (103.09) | 189.82 (143.99) | 158.24 (91.89) |
| Median (IQR) | 149.45 (139.74) | 204.53 (131.54) | 139.97 (127.87) | 204.53 (107.46) | 171.89 (145.06) | 215.58 (167.97) | 173.22 (114.79) | 173.11 (140.80) |
| Range | 0.07 - 1913.50 | 35.63 - 412.66 | 0.04 - 926.01 | 1.04 - 291.26 | 0.16 - 1186.13 | 0.23 - 310.41 | 0.56 - 1101.81 | 0.59 - 296.54 |

GFAP = Glial Fibrillary Acidic Protein; NFL = Neurofilament Light; IFNγ = Interferon Gamma; IL = Interleukin; MIP = Macrophage Inflammatory Protein; MCP = Monocyte Chemoattractant Protein; IP = IFNγ-induced Protein; TNF = Tumor Necrotic Factor; FGF-basic = Basic Fibroblast Growth Factor; PDGF = Platelet-derived Growth Factor

**Supplementary Table 3**. Summary of linear mixed model main effects assessing the longitudinal evolution of biomarkers as a function of traumatic intracranial findings. For each biomarker, main effects of group (CT+/CT- versus MRI+/MRI-), and the interaction of group by time are presented.

|  | **CT Imaging** | | **MRI Imaging** | |
| --- | --- | --- | --- | --- |
|  | **Group**  *p-value* | **Int. (Group x Time)**  *p-value* | **Group**  *p-value* | **Int. (Group x Time)**  *p-value* |
| GFAP ^†^ | **p = 0.003** | **p = 0.003** | p = 0.090 | **p < 0.001** |
| NFL ^†^ | **p = 0.009** | **p < 0.001** | **p = 0.008** | **p < 0.001** |
| Tau ^†^ | p = 0.208 | p = 0.255 | p = 0.336 | p = 0.027 |
| IFNγ: | p = 0.632 | p = 0.229 | p = 0.085 | p = 0.019 |
| IL-8 | p = 0.224 | p = 0.821 | p = 0.036 | p = 0.200 |
| Eotaxin ^†^ | p = 0.015 | p = 0.784 | **p = 0.001** | p = 0.373 |
| MIP-1β: | **p < 0.001** | p = 0.857 | **p < 0.001** | p = 0.099 |
| MCP-1 ^†^ | p = 0.653 | p = 0.241 | p = 0.220 | p = 0.086 |
| IP-10 ^†^ | **p < 0.001** | p = 0.458 | **p < 0.001** | p = 0.151 |
| IL-17A | p = 0.989 | p = 0.978 | p = 0.099 | p = 0.146 |
| IL-9 | p = 0.027 | p = 0.868 | **p = 0.003** | p = 0.047 |
| TNF | p = 0.192 | p = 0.681 | p = 0.031 | p = 0.111 |
| FGF-basic | p = 0.321 | p = 0.124 | p = 0.046 | **p = 0.004** |
| PDGF | p = 0.769 | p = 0.819 | p = 0.075 | p = 0.615 |
| IL-1ra ^†^ | p = 0.282 | p = 0.066 | p = 0.845 | p = 0.363 |

^†^ Indicates base-10 log transformed data. Significant p-values are bolded (α = 0.01).

Int. = Interaction; GFAP = Glial fibrillary acidic protein; NFL = Neurofilament light; IFNγ = Interferon Gamma; IL = Interleukin; MIP = Macrophage Inflammatory Protein; MCP = Monocyte Chemoattractant Protein; IP = IFNγ-induced Protein; TNF = Tumor Necrotic Factor; FGF-basic = Basic Fibroblast Growth Factor; PDGF = Platelet-derived Growth Factor

**Supplementary Table 4**. Summary of linear mixed model main effects assessing the longitudinal evolution of biomarkers as a function of traumatic intracranial findings, with sex assessed as a covariate of interest. For each biomarker, main effects of group (CT+/CT- versus MRI+/MRI-), the interaction of group by time, sex and the interaction of sex by group, are presented.

|  | **CT Imaging** | | | | **MRI Imaging** | | | |  |
| --- | --- | --- | --- | --- | --- | --- | --- | --- | --- |
|  | **Group**  *p-value* | **Int. (Group x Time)**  *p-value* | **Sex**  *p-value* | **Int. (Sex x Group)**  *p-value* | **Group**  *p-value* | **Int. (Group x Time)**  *p-value* | **Sex**  *p-value* | **Int. (Sex x Group)**  *p-value* | |
| GFAP ^†^ | **p = 0.002** | **p = 0.002** | p = 0.025 | p = 0.379 | p = 0.084 | **p < 0.001** | p = 0.045 | p = 0.010 | |
| NFL ^†^ | **p = 0.008** | **p < 0.001** | p = 0.428 | p = 0.055 | **p = 0.001** | **p < 0.001** | p = 0.550 | p = 0.107 | |
| Tau ^†^ | p = 0.171 | p = 0.251 | **p < 0.001** | p = 0.736 | p = 0.316 | p = 0.032 | **p < 0.001** | p = 0.811 | |
| IFNγ: | p = 0.667 | p = 0.226 | p = 0.921 | p = 0.478 | p = 0.086 | p = 0.020 | p = 0.757 | p = 0.042 | |
| IL-8 | p = 0.321 | p = 0.972 | p = 0.810 | p = 0.973 | p = 0.037 | p = 0.206 | p = 0.754 | p = 0.531 | |
| Eotaxin ^†^ | p = 0.014 | p = 0.771 | p = 0.024 | p = 0.855 | **p = 0.001** | p = 0.392 | p = 0.021 | p = 0.895 | |
| MIP-1β: | **p < 0.001** | p = 0.850 | p = 0.340 | p = 0.901 | **p < 0.001** | p = 0.099 | p = 0.180 | p = 0.734 | |
| MCP-1 ^†^ | p = 0.654 | p = 0.240 | p = 0.262 | p = 0.907 | p = 0.451 | p = 0.160 | p = 0.256 | p = 0.554 | |
| IP-10 ^†^ | **p < 0.001** | p = 0.483 | p = 0.576 | p = 0.298 | **p < 0.001** | p = 0.166 | p = 0.909 | p = 0.419 | |
| IL-17A | p = 0.974 | p = 0.978 | p = 0.407 | p = 0.943 | p = 0.098 | p = 0.155 | p = 0.256 | p = 0.323 | |
| IL-9 | p = 0.029 | p = 0.868 | p = 0.831 | p = 0.869 | **p = 0.003** | p = 0.048 | p = 0.577 | p = 0.481 | |
| TNF | p = 0.200 | p = 0.945 | p = 0.651 | p = 0.539 | p = 0.040 | p = 0.487 | p = 0.937 | p = 0.743 | |
| FGF-basic | p = 0.322 | p = 0.120 | p = 0.409 | p = 0.709 | p = 0.047 | **p = 0.004** | p = 0.283 | p = 0.403 | |
| PDGF | p = 0.768 | p = 0.861 | p = 0.048 | p = 0.589 | p = 0.073 | p = 0.602 | p = 0.020 | p = 0.730 | |
| IL-1ra ^†^ | p = 0.286 | p = 0.066 | p = 0.869 | p = 0.938 | p = 0.856 | p = 0.378 | p = 0.748 | p = 0.215 | |

^†^ Indicates base-10 log transformed data. Significant p-values are bolded (α = 0.01).

Int. = Interaction; GFAP = Glial fibrillary acidic protein; NFL = Neurofilament light; IFNγ = Interferon Gamma; IL = Interleukin; MIP = Macrophage Inflammatory Protein; MCP = Monocyte Chemoattractant Protein; IP = IFNγ-induced Protein; TNF = Tumor Necrotic Factor; FGF-basic = Basic Fibroblast Growth Factor; PDGF = Platelet-derived Growth Factor

**Supplementary Table 5:** Penalized regression coefficients from elastic net regression and error estimates from 1000 bootstrapped samples showing the combined utility of blood biomarkers for predicting patients with mTBI who were CT+ and/or MRI+ at each timepoint.

|  | **Admission** | | **2 weeks** | | **3 months** | | **12 months** | |
| --- | --- | --- | --- | --- | --- | --- | --- | --- |
|  | **Estimate** | **Percentage coefficient not 0** | **Estimate** | **Percentage coefficient not 0** | **Estimate** | **Percentage coefficient not 0** | **Estimate** | **Percentage coefficient not 0** |
| **CT Findings:** |  |  |  |  |  |  |  |  |
| GFAP | 0.21 | 88.00 | 0.07 | 79.90 | 0 | 88.70 | 0 | 74.20 |
| NFL | 0.27 | **94.20** | 0.26 | **92.60** | 0.36 | **95.70** | 0 | 80.50 |
| Tau | 0 | 58.60 | 0 | 73.30 | 0 | 80.20 | 0 | 73.20 |
| IFNγ | 0 | 65.30 | 0 | 75.90 | 0 | 64.90 | 0 | 56.50 |
| IL-8 | 0 | 65.60 | 0 | 52.70 | 0 | 56.70 | 0 | 52.90 |
| Eotaxin | -0.07 | 71.80 | -0.17 | **90.40** | 0 | 85.60 | 0 | 84.40 |
| MIP-1β | -0.22 | **92.30** | -0.53 | **99.90** | -0.20 | **92.80** | -0.12 | **93.80** |
| MCP-1 | 0 | 41.80 | 0 | 44.30 | 0 | 62.20 | 0 | 58.20 |
| IP-10 | -0.19 | **90.60** | -0.10 | 81.70 | -0.16 | **95.60** | -0.16 | **96.70** |
| IL-17A | 0 | 59.90 | 0 | 68.00 | 0 | 71.30 | 0 | 70.90 |
| IL-9 | 0 | 40.30 | 0 | 44.30 | 0 | 67.50 | 0 | 77.00 |
| TNF | 0 | 47.70 | 0 | 60.70 | 0 | 63.50 | 0 | 62.30 |
| FGF-basic | 0 | 44.70 | 0 | 49.90 | 0 | 52.90 | 0 | 64.00 |
| PDGF | 0 | 55.90 | 0 | 87.00 | 0 | 82.90 | 0 | 70.70 |
| IL-1ra | 0.03 | 78.60 | 0.15 | **95.90** | 0 | 61.00 | 0 | 78.10 |
| **MRI Findings:** |  |  |  |  |  |  |  |  |
| GFAP | 0.30 | **100.00** | 0.09 | 51.40 | 0 | 43.00 | 0 | 54.40 |
| NFL | 0.09 | 84.00 | 0.45 | **100.00** | 0.43 | **100.00** | 0 | 46.00 |
| Tau | 0 | 46.80 | 0 | 33.40 | 0 | 49.00 | 0 | 63.30 |
| IFNγ | 0 | 45.10 | 0 | 30.00 | 0 | 43.40 | 0 | 39.80 |
| IL-8 | 0 | 57.80 | 0 | 18.30 | 0 | 28.30 | 0 | 38.70 |
| Eotaxin | -0.06 | 89.90 | -0.09 | 74.80 | 0 | 76.50 | 0 | 80.50 |
| MIP-1β | -0.06 | **91.40** | -0.09 | 65.30 | -0.14 | 86.70 | -0.08 | 87.80 |
| MCP-1 | 0 | 33.20 | 0 | 25.30 | 0 | 37.20 | 0 | 47.20 |
| IP-10 | -0.11 | **95.50** | -0.08 | 72.20 | -0.03 | 80.00 | 0 | 75.10 |
| IL-17A | 0 | 34.00 | 0 | 13.10 | 0 | 38.70 | 0 | 44.50 |
| IL-9 | 0 | 37.50 | 0 | 26.80 | 0 | 53.20 | -0.10 | 86.80 |
| TNF | 0 | 41.00 | 0 | 20.30 | 0 | 46.30 | 0 | 49.00 |
| FGF-basic | 0 | 35.10 | 0 | 20.50 | 0 | 31.70 | 0 | 54.90 |
| PDGF | 0 | 46.80 | 0 | 26.40 | 0 | 40.90 | 0 | 47.00 |
| IL-1ra | 0 | 70.60 | 0 | 31.90 | 0 | 47.70 | 0 | 52.80 |

Estimate refers to the estimated penalized regression coefficient of the optimal model. Percentage coefficient not 0 refers to the percentage of times the variable was not set to 0 in the 1000 bootstrapped repetitions, i.e., in what proportion of models the variable showed an important effect on the outcome. A higher percentage indicates greater likelihood of a true population effect and is therefore a proxy for a confidence interval in this analysis. Percentages above 90% are bolded.

GFAP = Glial fibrillary acidic protein; NFL = Neurofilament light; IFNγ = Interferon Gamma; IL = Interleukin; MIP = Macrophage Inflammatory Protein; MCP = Monocyte Chemoattractant Protein; IP = IFNγ-induced Protein; TNF = Tumor Necrotic Factor; FGF-basic = Basic Fibroblast Growth Factor; PDGF = Platelet-derived Growth Factor

**Supplementary Table 6:** Area under the curve (AUCs), sensitivities, specificities and thresholds based on both Youden’s J statistic and sensitivity-maximized thresholds, showing the ability of individual biomarkers - at each timepoint - to discriminate patients with mTBI who were CT+ from CT-.

|  | **Youden’s J Statistic** | | |  | **Sensitivity-maximized** | | |
| --- | --- | --- | --- | --- | --- | --- | --- |
|  | **Sensitivity** | **Specificity** | **Threshold**  (pg/mL) | **AUC** [95% CI] | **Sensitivity** | **Specificity** | **Threshold**  (pg/mL) |
| **GFAP:** |  |  |  |  |  |  |  |
| Admission | 1.00 | 0.58 | 83.80 | 0.78 [0.69 – 0.87] | 1.00 | 0.58 | 83.80 |
| 2 weeks | 0.36 | 0.99 | 152.18 | 0.72 [0.55 – 0.89] | 1.00 | 0.25 | 33.73 |
| 3 months | 0.70 | 0.59 | 41.86 | 0.67 [0.49 – 0.85] | 1.00 | 0.24 | 29.20 |
| 12 months | 0.70 | 0.46 | 39.03 | 0.48 [0.30 – 0.65] | 1.00 | 0.008 | 95.44 |
| **NFL:** |  |  |  |  |  |  |  |
| Admission | 0.67 | 0.82 | 9.47 | 0.76 [0.62 – 0.90] | 1.00 | 0.12 | 3.26 |
| 2 weeks | 1.00 | 0.54 | 9.51 | 0.81 [0.70 – 0.92] | 1.00 | 0.54 | 9.51 |
| 3 months | 0.60 | 0.87 | 15.28 | 0.77 [0.61 – 0.92] | 1.00 | 0.36 | 5.23 |
| 12 months | 0.70 | 0.64 | 4.51 | 0.64 [0.46 – 0.82] | 1.00 | 0.07 | 9.79 |
| **Tau:** |  |  |  |  |  |  |  |
| Admission | 0.80 | 0.48 | 2.22 | 0.60 [0.45 – 0.74] | 1.00 | 0.05 | 0.94 |
| 2 weeks | 0.36 | 0.81 | 3.09 | 0.56 [0.37 – 0.76] | 1.00 | 0.08 | 1.36 |
| 3 months | 0.50 | 0.66 | 2.75 | 0.44 [0.25 – 0.63] | 1.00 | 0.008 | 0.41 |
| 12 months | 0.70 | 0.61 | 1.91 | 0.60 [0.40 – 0.80] | 1.00 | 0.07 | 3.86 |
| **IFNγ:** |  |  |  |  |  |  |  |
| Admission | 0.67 | 0.50 | 2.95 | 0.55 [0.39 – 0.70] | 1.00 | 0.007 | 0.02 |
| 2 weeks | 0.73 | 0.60 | 3.32 | 0.62 [0.45 – 0.79] | 1.00 | 0.08 | 0.11 |
| 3 months | 0.90 | 0.22 | 0.87 | 0.47 [0.31 – 0.63] | 1.00 | 0.02 | 0.04 |
| 12 months | 0.80 | 0.45 | 3.29 | 0.50 [0.36 – 0.63] | 1.00 | 0.13 | 0.20 |
| **IL-8:** |  |  |  |  |  |  |  |
| Admission | 0.80 | 0.46 | 4.94 | 0.52 [0.40 – 0.63] | 1.00 | 0.03 | 0.04 |
| 2 weeks | 0.82 | 0.44 | 5.98 | 0.53 [0.40 – 0.67] | 1.00 | 0.05 | 0.04 |
| 3 months | 1.00 | 0.22 | 17.96 | 0.51 [0.38 – 0.64] | 1.00 | 0.22 | 17.96 |
| 12 months | 0.90 | 0.25 | 3.90 | 0.50 [0.35 – 0.65] | 1.00 | 0.13 | 0.24 |
| **Eotaxin:** |  |  |  |  |  |  |  |
| Admission | 0.80 | 0.58 | 29.09 | 0.70 [0.58 – 0.81] | 1.00 | 0.33 | 37.85 |
| 2 weeks | 1.00 | 0.51 | 32.92 | 0.76 [0.65 – 0.87] | 1.00 | 0.51 | 32.92 |
| 3 months | 0.80 | 0.65 | 26.80 | 0.71 [0.57 – 0.85] | 1.00 | 0.35 | 38.93 |
| 12 months | 0.80 | 0.64 | 28.00 | 0.70 [0.56 – 0.84] | 1.00 | 0.34 | 41.25 |
| **MIP-1β:** |  |  |  |  |  |  |  |
| Admission | 0.80 | 0.75 | 78.94 | 0.79 [0.69 – 0.89] | 1.00 | 0.29 | 117.66 |
| 2 weeks | 0.82 | 0.70 | 91.96 | 0.79 [0.66 – 0.91] | 1.00 | 0.35 | 121.26 |
| 3 months | 0.70 | 0.81 | 82.75 | 0.79 [0.65 – 0.93] | 1.00 | 0.35 | 119.18 |
| 12 months | 0.80 | 0.86 | 75.22 | 0.81 [0.63 – 0.98] | 1.00 | 0.11 | 139.63 |
| **MCP-1:** |  |  |  |  |  |  |  |
| Admission | 0.80 | 0.54 | 12.73 | 0.57 [0.46 – 0.69] | 1.00 | 0.22 | 19.63 |
| 2 weeks | 1.00 | 0.44 | 14.30 | 0.64 [0.54 – 0.75] | 1.00 | 0.44 | 14.30 |
| 3 months | 0.90 | 0.50 | 14.18 | 0.69 [0.54 – 0.84] | 1.00 | 0.20 | 22.13 |
| 12 months | 0.80 | 0.64 | 12.74 | 0.67 [0.54 – 0.80] | 1.00 | 0.25 | 19.78 |
| **IP-10:** |  |  |  |  |  |  |  |
| Admission | 0.67 | 0.78 | 180.24 | 0.75 [0.63 – 0.88] | 1.00 | 0.24 | 436.88 |
| 2 weeks | 0.91 | 0.54 | 298.50 | 0.76 [0.64 – 0.88] | 1.00 | 0.39 | 360.36 |
| 3 months | 0.70 | 0.85 | 184.78 | 0.79 [0.65 – 0.93] | 1.00 | 0.24 | 455.88 |
| 12 months | 0.70 | 0.89 | 160.50 | 0.78 [0.61 – 0.95] | 1.00 | 0.27 | 423.15 |
|  |  |  |  |  |  |  |  |
| **IL-17A:** |  |  |  |  |  |  |  |
| Admission | 0.67 | 0.58 | 11.54 | 0.58 [0.46 – 0.71] | 1.00 | 0.20 | 0.12 |
| 2 weeks | 0.91 | 0.40 | 10.68 | 0.59 [0.46 – 0.72] | 1.00 | 0.13 | 0.15 |
| 3 months | 0.80 | 0.40 | 18.88 | 0.49 [0.34 – 0.64] | 1.00 | 0.15 | 27.12 |
| 12 months | 0.70 | 0.50 | 16.48 | 0.52 [0.35 – 0.68] | 1.00 | 0.15 | 25.48 |
| **IL-9:** |  |  |  |  |  |  |  |
| Admission | 0.87 | 0.66 | 45.42 | 0.70 [0.61 – 0.79] | 1.00 | 0.29 | 65.78 |
| 2 weeks | 0.82 | 0.69 | 46.30 | 0.72 [0.60 – 0.84] | 1.00 | 0.34 | 70.26 |
| 3 months | 0.80 | 0.73 | 48.56 | 0.72 [0.56 – 0.88] | 1.00 | 0.21 | 84.15 |
| 12 months | 0.80 | 0.72 | 50.02 | 0.74 [0.56 – 0.91] | 1.00 | 0.20 | 85.56 |
| **TNF:** |  |  |  |  |  |  |  |
| Admission | 0.87 | 0.41 | 38.90 | 0.56 [0.44 – 0.69] | 1.00 | 0.13 | 58.36 |
| 2 weeks | 0.55 | 0.61 | 38.26 | 0.49 [0.33 – 0.64] | 1.00 | 0.05 | 12.45 |
| 3 months | 0.80 | 0.49 | 40.64 | 0.60 [0.41 – 0.78] | 1.00 | 0.08 | 70.96 |
| 12 months | 0.80 | 0.46 | 41.66 | 0.61 [0.43 – 0.79] | 1.00 | 0.11 | 63.17 |
| **FGF-basic:** |  |  |  |  |  |  |  |
| Admission | 0.80 | 0.43 | 19.94 | 0.53 [0.43 – 0.63] | 1.00 | 0.19 | 5.02 |
| 2 weeks | 0.82 | 0.40 | 40.41 | 0.53 [0.39 – 0.67] | 1.00 | 0.17 | 57.42 |
| 3 months | 0.80 | 0.53 | 36.80 | 0.55 [0.41 – 0.68] | 1.00 | 0.16 | 65.09 |
| 12 months | 0.90 | 0.45 | 46.41 | 0.58 [0.43 – 0.73] | 1.00 | 0.14 | 70.76 |
| **PDGF:** |  |  |  |  |  |  |  |
| Admission | 0.60 | 0.58 | 223.21 | 0.53 [0.38 – 0.67] | 1.00 | 0.06 | 940.64 |
| 2 weeks | 0.70 | 0.56 | 369.94 | 0.57 [0.37 – 0.76] | 1.00 | 0.10 | 87.92 |
| 3 months | 0.90 | 0.31 | 241.75 | 0.58 [0.40 – 0.75] | 1.00 | 0.12 | 142.22 |
| 12 months | 0.70 | 0.59 | 325.11 | 0.55 [0.35 – 0.75] | 1.00 | 0.06 | 939.38 |
|  |  |  |  |  |  |  |  |
| **IL-1ra** |  |  |  |  |  |  |  |
| Admission | 0.73 | 0.60 | 185.78 | 0.61 [0.46 – 0.76] | 1.00 | 0.08 | 28.98 |
| 2 weeks | 0.82 | 0.59 | 168.64 | 0.68 [0.53 – 0.83] | 1.00 | 0.09 | 7.50 |
| 3 months | 0.70 | 0.70 | 228.97 | 0.57 [0.37 – 0.77] | 1.00 | 0.04 | 23.40 |
| 12 months | 0.90 | 0.41 | 146.45 | 0.63 [0.45 – 0.81] | 1.00 | 0.04 | 23.40 |

AUC = Area Under the Curve; ROC = Receiver Operating Curve; 95% CI = the 95% confidence interval of the estimated AUC; mTBI = mild traumatic brain injury;

GFAP = Glial fibrillary acidic protein; NFL = Neurofilament light; IFNγ = Interferon Gamma; IL = Interleukin; MIP = Macrophage Inflammatory Protein; MCP = Monocyte Chemoattractant Protein; IP = IFNγ-induced Protein; TNF = Tumor Necrotic Factor; FGF-basic = Basic Fibroblast Growth Factor; PDGF = Platelet-derived Growth Factor

**Supplementary Table 7:** Area under the curve (AUCs), sensitivities, specificities and thresholds based on both Youden’s J Statistic and sensitivity-maximized thresholds, showing the ability of individual biomarkers - at each timepoint - to discriminate patients with mTBI who were MRI+ from MRI-.

|  | **Youden’s J Statistic** | | |  | **Sensitivity-maximized** | | |
| --- | --- | --- | --- | --- | --- | --- | --- |
|  | **Sensitivity** | **Specificity** | **Threshold**  (pg/mL) | **AUC** [95% CI] | **Sensitivity** | **Specificity** | **Threshold**  (pg/mL) |
| **GFAP:** |  |  |  |  |  |  |  |
| Admission | 0.92 | 0.63 | 83.80 | 0.82 [0.73 – 0.91] | 1.00 | 0.14 | 31.40 |
| 2 weeks | 0.53 | 0.94 | 97.24 | 0.75 [0.62 – 0.89] | 1.00 | 0.06 | 18.06 |
| 3 months | 0.53 | 0.66 | 43.73 | 0.56 [0.41 – 0.72] | 1.00 | 0.03 | 11.79 |
| 12 months | 0.67 | 0.62 | 34.42 | 0.61 [0.46 – 0.76] | 1.00 | 0.02 | 95.44 |
| **NFL:** |  |  |  |  |  |  |  |
| Admission | 0.67 | 0.74 | 7.93 | 0.73 [0.62 – 0.84] | 1.00 | 0.13 | 3.26 |
| 2 weeks | 0.74 | 0.90 | 44.97 | 0.89 [0.83 – 0.96] | 1.00 | 0.58 | 9.51 |
| 3 months | 0.68 | 0.92 | 15.28 | 0.86 [0.77 – 0.95] | 1.00 | 0.39 | 5.23 |
| 12 months | 0.94 | 0.23 | 7.62 | 0.54 [0.41 – 0.67] | 1.00 | 0.09 | 9.76 |
| **Tau:** |  |  |  |  |  |  |  |
| Admission | 0.54 | 0.67 | 2.81 | 0.62 [0.50 – 0.74] | 1.00 | 0.05 | 0.94 |
| 2 weeks | 0.68 | 0.48 | 2.40 | 0.56 [0.42 – 0.70] | 1.00 | 0.08 | 1.36 |
| 3 months | 0.89 | 0.18 | 1.45 | 0.44 [0.32 – 0.56] | 1.00 | 0.007 | 0.41 |
| 12 months | 0.78 | 0.53 | 2.07 | 0.61 [0.48 – 0.75] | 1.00 | 0.08 | 3.85 |
| **IFNγ:** |  |  |  |  |  |  |  |
| Admission | 0.61 | 0.51 | 2.95 | 0.50 [0.37 – 0.62] | 1.00 | 0.00 | – |
| 2 weeks | 0.68 | 0.59 | 3.14 | 0.57 [0.45 – 0.69] | 1.00 | 0.11 | 0.11 |
| 3 months | 0.95 | 0.32 | 4.96 | 0.61 [0.49 – 0.74] | 1.00 | 0.24 | 5.81 |
| 12 months | 1.00 | 0.30 | 6.50 | 0.62 [0.51 – 0.74] | 1.00 | 0.30 | 6.50 |
| **IL-8:** |  |  |  |  |  |  |  |
| Admission | 0.74 | 0.47 | 4.94 | 0.50 [0.40 – 0.60] | 1.00 | 0.03 | 0.04 |
| 2 weeks | 0.68 | 0.54 | 6.60 | 0.49 [0.36 – 0.61] | 1.00 | 0.00 | – |
| 3 months | 0.95 | 0.33 | 13.41 | 0.60 [0.48 – 0.71] | 1.00 | 0.24 | 17.96 |
| 12 months | 0.61 | 0.67 | 6.27 | 0.64 [0.52 – 0.76] | 1.00 | 0.24 | 14.03 |
| **Eotaxin:** |  |  |  |  |  |  |  |
| Admission | 0.87 | 0.57 | 30.56 | 0.76 [0.67 – 0.84] | 1.00 | 0.38 | 37.79 |
| 2 weeks | 0.89 | 0.54 | 32.92 | 0.74 [0.63 – 0.85] | 1.00 | 0.15 | 58.84 |
| 3 months | 0.74 | 0.70 | 26.80 | 0.73 [0.63 – 0.83] | 1.00 | 0.31 | 44.39 |
| 12 months | 0.72 | 0.71 | 28.00 | 0.74 [0.64 – 0.84] | 1.00 | 0.42 | 41.25 |
| **MIP-1β:** |  |  |  |  |  |  |  |
| Admission | 0.74 | 0.75 | 82.25 | 0.75 [0.65 – 0.86] | 1.00 | 0.24 | 122.70 |
| 2 weeks | 0.74 | 0.69 | 91.96 | 0.72 [0.59 – 0.85] | 1.00 | 0.17 | 131.66 |
| 3 months | 0.68 | 0.83 | 85.60 | 0.79 [0.67 – 0.90] | 1.00 | 0.25 | 128.005 |
| 12 months | 0.78 | 0.84 | 81.58 | 0.81 [0.68 – 0.95] | 1.00 | 0.04 | 152.11 |
| **MCP-1:** |  |  |  |  |  |  |  |
| Admission | 0.78 | 0.55 | 12.73 | 0.60 [0.51 – 0.69] | 1.00 | 0.24 | 19.63 |
| 2 weeks | 0.94 | 0.45 | 14.30 | 0.65 [0.56 – 0.75] | 1.00 | 0.36 | 16.44 |
| 3 months | 0.89 | 0.51 | 14.18 | 0.73 [0.62 – 0.83] | 1.00 | 0.22 | 22.13 |
| 12 months | 0.83 | 0.68 | 12.74 | 0.74 [0.64 – 0.85] | 1.00 | 0.28 | 19.78 |
| **IP-10:** |  |  |  |  |  |  |  |
| Admission | 0.74 | 0.75 | 200.28 | 0.77 [0.67 – 0.86] | 1.00 | 0.24 | 436.88 |
| 2 weeks | 0.95 | 0.48 | 311.36 | 0.73 [0.64 – 0.83] | 1.00 | 0.38 | 360.36 |
| 3 months | 0.84 | 0.60 | 271.74 | 0.75 [0.64 – 0.86] | 1.00 | 0.25 | 491.43 |
| 12 months | 0.67 | 0.79 | 219.22 | 0.75 [0.64 – 0.86] | 1.00 | 0.30 | 424.40 |
|  |  |  |  |  |  |  |  |
| **IL-17A:** |  |  |  |  |  |  |  |
| Admission | 0.57 | 0.52 | 10.70 | 0.49 [0.37 – 0.61] | 1.00 | 0.00 | – |
| 2 weeks | 0.74 | 0.49 | 11.55 | 0.52 [0.40 – 0.63] | 1.00 | 0.08 | 0.06 |
| 3 months | 0.84 | 0.41 | 18.88 | 0.60 [0.49 – 0.72] | 1.00 | 0.17 | 27.12 |
| 12 months | 0.78 | 0.52 | 16.48 | 0.65 [0.51 – 0.79] | 1.00 | 0.15 | 25.96 |
| **IL-9:** |  |  |  |  |  |  |  |
| Admission | 0.91 | 0.56 | 53.28 | 0.70 [0.61 – 0.78] | 1.00 | 0.24 | 69.53 |
| 2 weeks | 0.74 | 0.69 | 46.30 | 0.68 [0.57 – 0.78] | 1.00 | 0.24 | 75.50 |
| 3 months | 0.74 | 0.73 | 48.56 | 0.74 [0.63 – 0.86] | 1.00 | 0.23 | 84.15 |
| 12 months | 0.72 | 0.81 | 44.20 | 0.79 [0.67 – 0.91] | 1.00 | 0.22 | 85.56 |
| **TNF:** |  |  |  |  |  |  |  |
| Admission | 0.91 | 0.41 | 38.90 | 0.59 [0.49 – 0.68] | 1.00 | 0.13 | 58.36 |
| 2 weeks | 0.89 | 0.30 | 44.76 | 0.53 [0.40 – 0.66] | 1.00 | 0.11 | 65.48 |
| 3 months | 0.47 | 0.82 | 26.44 | 0.67 [0.54 – 0.80] | 1.00 | 0.08 | 70.96 |
| 12 months | 0.72 | 0.68 | 33.64 | 0.71 [0.58 – 0.83] | 1.00 | 0.14 | 63.17 |
| **FGF-basic:** |  |  |  |  |  |  |  |
| Admission | 0.74 | 0.43 | 18.92 | 0.50 [0.40 – 0.60] | 1.00 | 0.006 | 0.08 |
| 2 weeks | 0.89 | 0.39 | 40.41 | 0.57 [0.46 – 0.69] | 1.00 | 0.15 | 57.42 |
| 3 months | 0.84 | 0.51 | 38.13 | 0.64 [0.53 – 0.76] | 1.00 | 0.17 | 65.09 |
| 12 months | 0.83 | 0.53 | 38.73 | 0.67 [0.55 – 0.79] | 1.00 | 0.14 | 70.76 |
| **PDGF:** |  |  |  |  |  |  |  |
| Admission | 0.70 | 0.46 | 302.46 | 0.54 [0.41 – 0.66] | 1.00 | 0.07 | 940.005 |
| 2 weeks | 0.50 | 0.57 | 369.94 | 0.47 [0.33 – 0.62] | 1.00 | 0.04 | 38.61 |
| 3 months | 0.63 | 0.67 | 293.31 | 0.63 [0.50 – 0.77] | 1.00 | 0.10 | 817.82 |
| 12 months | 0.72 | 0.61 | 325.11 | 0.64 [0.51 – 0.78] | 1.00 | 0.06 | 939.38 |
|  |  |  |  |  |  |  |  |
| **IL-1ra** |  |  |  |  |  |  |  |
| Admission | 0.65 | 0.62 | 180.53 | 0.56 [0.44 – 0.69] | 1.00 | 0.08 | 28.98 |
| 2 weeks | 0.63 | 0.70 | 190.16 | 0.60 [0.46 – 0.74] | 1.00 | 0.05 | 0.96 |
| 3 months | 0.53 | 0.67 | 213.24 | 0.45 [0.30 – 0.60] | 1.00 | 0.007 | 0.20 |
| 12 months | 0.33 | 0.84 | 95.84 | 0.53 [0.38 – 0.69] | 1.00 | 0.12 | 296.74 |

AUC = Area Under the Curve; AUCs >= 0.80 are bolded. ROC = Receiver Operating Curve; 95% CI = the 95% confidence interval of the estimated AUC; mTBI = mild traumatic brain injury; GFAP = Glial fibrillary acidic protein; NFL = Neurofilament light; IFNγ = Interferon Gamma; IL = Interleukin; MIP = Macrophage Inflammatory Protein; MCP = Monocyte Chemoattractant Protein; IP = IFNγ-induced Protein; TNF = Tumor Necrotic Factor; FGF-basic = Basic Fibroblast Growth Factor; PDGF = Platelet-derived Growth Factor
